# A Virome Scanning of Saffron (*Crocus sativus* L.) at the National Scale in Iran Using High-Throughput Sequencing Technologies

**DOI:** 10.3390/v17081079

**Published:** 2025-08-04

**Authors:** Hajar Valouzi, Akbar Dizadji, Alireza Golnaraghi, Seyed Alireza Salami, Nuria Fontdevila Pareta, Serkan Önder, Ilhem Selmi, Johan Rollin, Chadi Berhal, Lucie Tamisier, François Maclot, Long Wang, Rui Zhang, Habibullah Bahlolzada, Pierre Lefeuvre, Sébastien Massart

**Affiliations:** 1 Department of Plant Protection, Faculty of Agriculture, University College of Agriculture & Natural Resources, University of Tehran, Karaj 7787131587, Iran; hvalouzi@gmail.com; 2 Department of Plant Protection, Faculty of Agricultural Sciences and Food Industries, Science and Research Branch, Islamic Azad University, Tehran 1477893855, Iran; 3 Department of Earth, Ocean & Atmospheric Sciences, University of British Columbia, Vancouver, BC V6T 1Z4, Canada; 4 Department of Biodiversity, BoomZista Institute, Vancouver, BC V6M 3W4, Canada; 5 Department of Horticultural Science, Faculty of Agriculture, University of Tehran, Karaj 7787131587, Iran; asalami@ut.ac.ir; 6 Integrated and Urban Plant Pathology Laboratory, TERRA Teaching and Research Centre, Gembloux Agro-Bio Tech, University of Liège, 5030 Gembloux, Belgium; nuria.fontdevilapareta@agroscope.admin.ch (N.F.P.); onderserkan@gmail.com (S.Ö.); ilhemselmi@hotmail.com (I.S.); johan.rollin@outlook.fr (J.R.); chadi.berhal@gmail.com (C.B.);; 7 Plant Protection Department, Agroscope, 1260 Nyon, Switzerland; 8 Department of Plant Protection, Faculty of Agriculture, Eskişehir Osmangazi University, Eskişehir 26160, Türkiye; 9 DNAVision, 6041 Gosselies, Belgium; 10 GAFL (Genetics and Improvement of Fruit and Vegetables), INRAE, Montfavet, 84140 Avignon, France; lucie.tamisier@inrae.fr; 11 UMR 1332 Biologie du Fruit et Pathologie (BFP), INRAE, Department of Environmental Sciences, University of Bordeaux, CS20032, 33882 Villenave d’Ornon Cedex, France; 12 Archaeal Biology Center, Institute for Advanced Study, Shenzhen University, Shenzhen 518060, China; kodragon@szu.edu.cn (L.W.); ruizhang@szu.edu.cn (R.Z.); 13 Shenzhen Key Laboratory of Marine Microbiome Engineering, Institute for Advanced Study, Shenzhen University, Shenzhen 518060, China; 14 Plant Protection Department, Faculty of Agriculture, Bamyan University, Bamyan 1601, Afghanistan; habib_bahlol@bu.edu.af; 15 UMR PVBMT (Plant Populations and Bio-Aggressors in Tropical Ecosystems), CIRAD, 97410 Saint-Pierre, La Ré-union, France; pierre.lefeuvre@cirad.fr

**Keywords:** *Crocus sativus*, saffron, virome, high-throughput sequencing, VANA, virus diversity, certification, virus surveillance

## Abstract

Saffron (*Crocus sativus* L.) is a vegetatively propagated crop of high economic and cultural value, potentially affected by viral infections that may impact its productivity. Despite Iran’s dominance in global saffron production, knowledge of its virome remains limited. In this study, we conducted the first nationwide virome survey of saffron in Iran employing a high-throughput sequencing (HTS) approach on pooled samples obtained from eleven provinces in Iran and one location in Afghanistan. Members of three virus families were detected—*Potyviridae* (*Potyvirus*), *Solemoviridae* (*Polerovirus*), and *Geminiviridae* (*Mastrevirus*)—as well as one satellite from the family *Alphasatellitidae* (*Clecrusatellite*). A novel *Potyvirus*, tentatively named saffron Iran virus (SaIRV) and detected in three provinces, shares less than 68% nucleotide identity with known Potyvirus species, thus meeting the ICTV criteria for designation as a new species. Genetic diversity analyses revealed substantial intrapopulation SNP variation but no clear geographical clustering. Among the two wild Crocus species sampled, only *Crocus speciosus* harbored turnip mosaic virus. Virome network and phylogenetic analyses confirmed widespread viral circulation likely driven by corm-mediated propagation. Our findings highlight the need for targeted certification programs and biological characterization of key viruses to mitigate potential impacts on saffron yield and quality.

## 1. Introduction

The saffron plant (*Crocus sativus* L., Iridaceae family), of which the famous spice is derived from its stigma, is vegetatively propagated through corms [1,2]. Often referred to as “Red gold”, saffron is renowned as the world’s most expensive spice, deriving its high value from its distinct color, taste, and aroma [3,4,5]. Utilized since ancient times by Iranians, saffron serves various purposes, such as boasting medicinal properties, enhancing flavors, and adding color to food [6]. Over time, its use has expanded across different cultures and countries, finding applications in modern pharmaceutical and cosmetic industries [7,8,9]. Iran is the primary global producer of saffron, contributing approximately 300 tons (90% of the production) to the world market [10]. Nevertheless, the yield of saffron production in Iran is significantly lower (3.5 kg/ha) compared to other producing countries like Spain (14 kg/ha) [4]. Saffron plants face persistent threats from a wide range of pathogens, including viruses [11]. A total of 20 virus species from eight families (*Alphaflexiviridae*, *Betaflexiviridae*, *Botourmiaviridae*, *Geminiviridae*, *Phenuiviridae*, *Potyviridae*, *Solemoviridae*, and *Tombusviridae*) have been reported so far in saffron from China, Hungary, India, Iran, Italy, Spain, and the Netherlands [12,13,14,15,16,17,18,19,20]. Among these, saffron yellow mosaic virus (SYMV), saffron betaflexivirus 1 (SaBV-1), saffron dwarf virus (SaDV), and saffron Iran virus (SaIR) have been discovered in the past two years using high-throughput sequencing technologies [18,20,21,22,23]. Although no comprehensive studies have explored the impact of these viruses on saffron production, some reports discussed the influence of viral infections on saffron [20]. Studies on saffron latent virus (SaLV) suggest that it affects saffron quality by altering secondary metabolite levels, including a decrease in the contents of picrocrocin, safranal, and some crocetin esters [24,25]. Accordingly, inoculation of cucumber mosaic virus (CMV) on saffron plants led to the highest reduction in crocin and safranal contents [26].

The vegetative propagation of saffron is an important risk factor for disease propagation to new locations, which could incur significant costs and impact trade negatively [27,28]. Therefore, improving the phytosanitary quality of saffron during the transfer and propagation of corms from source to destination is crucial. For this purpose, a certification scheme can be proposed for regulated non-quarantine organisms as well as quarantine measures, including systematic testing, trade limitation, controlled handling protocols, strict monitoring of corm health, and proper disinfection procedures for quarantine organisms. The very first basis for proposing appropriate phytosanitary measures is to know the geographical spreads of the viruses infecting the crop. Therefore, increasing knowledge about the saffron virome not only contributes to understanding its potential effects on crop productivity [20], but also strengthens quarantine efforts through improved virus detection strategies. Utilizing broad-spectrum detection tests such as high-throughput sequencing (HTS) technology enhances virus identification on a larger scale, further supporting effective quarantine programs [29].

In the last decade, HTS has emerged as a crucial tool for detecting viral diversity in various plants, especially cultivated crops. Multiple studies have demonstrated its strong potential for detecting both known and unknown viruses on a global scale and across extensive plant communities [30,31,32,33]. Furthermore, HTS has proven useful in identifying viruses in wild plants that may serve as reservoirs for viruses affecting major crops [34,35]. High-throughput sequencing (HTS) offers new opportunities for routine diagnostics, notably the ability to monitor viral presence across regions through large-scale surveillance programs [36,37]. To keep such programs cost-effective, sample pooling is often employed, although this can reduce analytical sensitivity. This limitation can be mitigated through viral enrichment strategies prior to sequencing. Various library preparation methods can be utilized as initial inputs for HTS, including total RNA, small RNA, double-stranded RNA (dsRNA), Rolling Circle Amplification (RCA), and Virion-Associated Nucleic Acids (VANA), the latter three enriching specifically the sample in virus nucleic acids [38,39,40]. For instance, the VANA approach is capable of detecting both DNA and RNA viruses simultaneously and can facilitate the reconstruction of more-complete viral genomes [39,41]. RCA can enrich circular DNA viruses through exponential amplification, providing a template for large-scale DNA sequencing [40,42]. The application of these enrichment methods, coupled with bioinformatic analysis, allowed sensitive detection, even on pooled samples, and the reconstruction of complete or nearly complete genomes [41].

Iran is the leading global producer and exporter of saffron, a crop of high economic importance but low yield. Despite its significance, limited data exist on viral infections in saffron. To address this gap, we conducted a nationwide virome survey using VANA applied to pooled saffron plants, incorporating internal and external alien controls, followed by HTS and in-depth bioinformatic analysis.

## 2. Materials and Methods

### 2.1. Sample Collection

**Figure 1:**
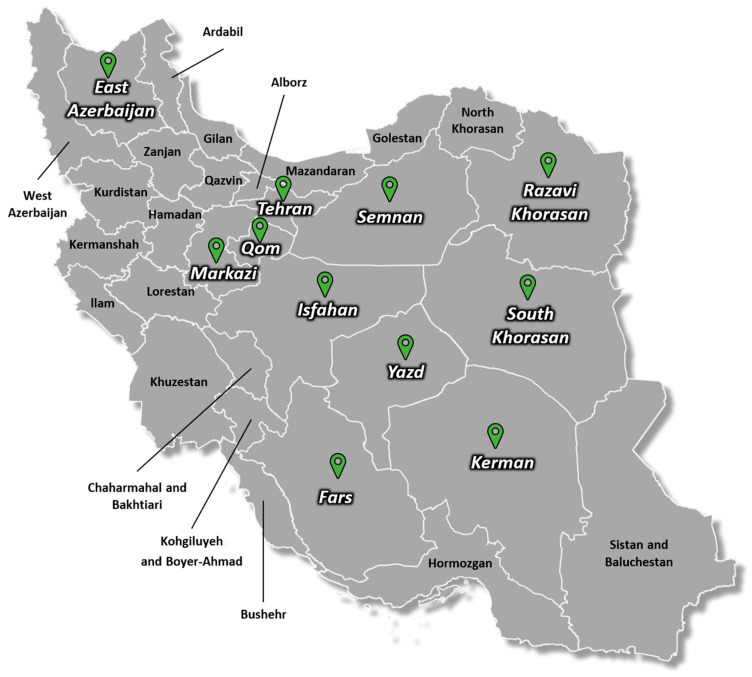
Map of Iran indicating the provinces from which samples were collected during the study. The sampled provinces include East Azerbaijan, Fars, Isfahan, Kerman, Razavi Khorasan, South Khorasan, Markazi, Qom, Semnan, Tehran, and Yazd. Sampling was conducted in 2022 as part of a virome survey targeting saffron.

Saffron leaves were collected during the winter of 2022 from the experimental site at the University of Tehran, Karaj, Iran. These leaves corresponded to corms originating from twelve distinct natural sites visited over several years, spanning eleven provinces in Iran and Bamyan province in Afghanistan, following a previously published strategy [43]. The corms were planted in the experimental station of the university experimental site where the aphid population was monitored and no significant aphid population was observed (only a few individuals in winter). The original collection sites included the following: eight sites from central regions (Isfahan, Markazi, Qom, Semnan, Tehran, Yazd), six sites from eastern regions (Razavi Khorasan and South Khorasan), four from southern regions (Fars and Kerman), and one from a western region (East Azerbaijan) (Figure 1). These sites were strategically selected to ensure broad geographic representation across the country. Additionally, two wild saffron species, *Crocus caspius* and *C. speciosus*, were sampled from the Botanical Garden of the University of Tehran, Karaj, Iran. In total, 1100 samples were collected, ranging from 50 to 150 samples per site/geographical origin (details provided in Table S1). From these, 22 pooled samples (designated as H), each consisting of 50 individual plants, were prepared. These included a mix of symptomatic (e.g., mosaic and mottling) and asymptomatic plants, with example images available in Supplementary Figure S1. The sites with 150 samples represent the main saffron-producing areas in Iran. Each pooled sample was weighed to 10 g (comprising 200 mg from each individual sample), then lyophilized, and stored at −20 °C until further use.

The external alien control (EAC) corresponded to a bean sample infected with three strains of *Phaseolus vulgaris* endornavirus (PvEV-1, PvEV-2, and PvEV-3; family *Endornaviridae*). The EAC contained a target (in this case, a virus) from the same group as the target organism(s) but absent from the saffron samples to monitor cross-contamination between samples. This was achieved by checking for the presence of the alien virus (PvEV-1, -2, and -3) in the saffron samples and, conversely, for any saffron-associated virus in the EAC [44]. Furthermore, internal positive controls (IPCs) were incorporated into each sample during HTS testing. This IPC was used to monitor the detection limit within each sample. Ideally, the chosen sequences for the IPC were consistently present at low levels within the analyzed matrix but above the detection threshold [44]. For this purpose, a banana sample infected at low concentration with the banana bract mosaic virus (BBrMV; *Potyviridae*) was used.

### 2.2. Virion-Associated Nucleic Acids—VANA

Viral particle enrichment was carried out for each pooled sample using the VANA extraction protocol [45]. First, 50 mL of Hanks’ buffered salt solution (HBSS, made up of 137 mM NaCl, 5.4 mM KCl, 0.25 mM Na_2_HPO_4_, 0.1 g glucose, 0.44 mM KH_2_PO_4_, 1.3 mM CaCl_2_, 1.0 mM MgSO_4_, and 4.2 mM NaHCO_3_) was mixed with 10 g of lyophilized plant tissue (saffron samples and bean as external control), using a tissue homogenizer. An alien external control of bean infected by three *Phaseolus vulgaris* endornaviruses (PvEV-1, -2, and -3) was processed in parallel to monitor potential cross-contaminations between samples [44]. The homogenized plant extract was centrifuged at 8000 g for 10 min, prior to being filtered through a 0.45 μm sterile syringe filter. An ultracentrifuge tube was filled with the supernatant. After ultracentrifugation, 0.2 mL (1:50) of the IPC was added to every tube [46]. A sucrose cushion was then placed at the tube’s bottom, consisting of 3 mL of 30% sucrose in 0.2 M potassium phosphate at pH 7.0. The extract was then centrifuged at 144,000× *g* for 2 h at 4 °C. The pellet was then resuspended in 1.5 mL of HBSS and kept overnight at 4 °C. Two hundred microliters of the suspension were digested with 15 U of bovine pancreatic DNase I, and 1.9 U of bovine pancreas RNase A (Euromedex, Souffelweyersheim, France), for 90 min at 37 °C. Then, through the use of the PureLink Viral RNA/DNA Mini Kit (Thermo Fisher Scientific, Merelbeke-Melle, Belgium), encapsidated nucleic acids were extracted in accordance with the manufacturer’s instructions. Library preparation was performed following the protocol by Palanga et al., as adapted by Maclot et al. [35,47]. Briefly, dodecamers and Superscript III (Invitrogen, Waltham, MA, USA) were used to create the viral cDNA, followed by priming and extension by Klenow polymerase, and were amplified with a multiplex identifier (MID) linker unique to each sample [48]. Two libraries were prepared, each containing eleven samples plus one alien external control, all tagged with twelve MIDs. Following the manufacturer’s instructions, the DNA product obtained from the sample was cleaned using the Nucleospin Gel and PCR clean-up (Macherey-Nagel, Düren, Germany). Libraries were prepared using the Illumina TruSeq PCR-Free library preparation kit, and the high-throughput sequencing was conducted on the NovaSeq 6000 platform with 2 × 150 nt reads at the Center of Biomedical Research of Liège University (GIGA, Liège, Belgium).

### 2.3. Rolling Circle Amplification—RCA

The Illustra™ TempliPhi 100 Amplification Kit (GE Healthcare, Amersham, UK) was used for Rolling Circle Amplification (RCA) on the pooled samples, each consisting of material from six individual plants. These pools originated from a total of 102 individual samples analyzed using the VANA approach, and the procedure was carried out following the manufacturer’s instructions. In this step, 1 μL of DNA extracted from saffron leaf tissue was added to 5 μL of sample buffer. The mixture was heated to 95 °C for three minutes. Then it was cooled, combined with 5 μL of reaction buffer and 0.2 μL of enzyme mix, and incubated at 30 °C for 18 h. At the end of this incubation, the DNA polymerase was inactivated by heating at 65 °C for 10 min. The resulting samples were then used for library construction, followed by high-throughput sequencing on the NovaSeq 6000 platform.

### 2.4. HTS Data Analysis and Genome Sequence Generation

Read quality was assessed using FastQC version 0.11.5 (https://www.bioinformatics.babraham.ac.uk/projects/fastqc, accessed on 19 August 2022). Raw reads were demultiplexed according to their internal MID linkers and subsequently removed at this stage [48,49], using commands available in the GitHub repository (V1.0)—*demultiplexing: diagnostic existing demultiplexing issue in metagenomic virus* [50]—executed in a Linux virtual desktop environment (MobaXterm Personal Edition v21.5). After demultiplexing, there were four groups of reads: reads with no MID in the pair (discarded), reads with the same MID for both reads (referred to as “both”, kept and considered as highest reliability), reads with one MID on the two reads (referred to as “single”, kept, although with risk of chimera), and reads with different MIDs for both reads (discarded as chimera). Reads were trimmed using BBDuK (ver 38.84), with low-quality bases removed from both ends if they fell below a minimum quality threshold of 32, and reads shorter than 50 bp were discarded. Then the reads were paired and merged and duplicates were removed using dedupe duplicate read remover (ver 38.37) in Geneious Prime (ver 2020.2) (https://www.geneious.com/, accessed on 19 August 2022). Reads were de novo-assembled into contigs using SPAdes assembler (ver 3.13.0) on Geneious Prime (ver 2020.2) and rnaviralSPADES on Galaxy (https://usegalaxy.eu, accessed on 19 August 2022). Potential contigs were identified by tBLASTx against the viral RefSeq database (version 1.1, downloaded on 25 March 2023 from the National Center for Biotechnology Information—NCBI). To extend the potential viral contigs identified, an iterative mapping of cleaned reads was carried out in Geneious Prime 2020.2 using the Geneious algorithm, with custom sensitivity, a maximum gap size of 15, and maximum mismatches based on the ICTV demarcation criteria for each virus. Species demarcation criteria and mismatch thresholds were applied according to ICTV guidelines: Potyvirus species were defined by <76% nucleotide identity across complete ORF sequences (24% mismatch threshold); Mastrevirus by <78% genome-wide identity to recognized species (22% mismatch threshold); Alphasatellite by <88% pairwise identity (12% mismatch threshold); and Polerovirus by >10% amino acid divergence in any gene product (10% mismatch threshold). For circular viruses, RCA-generated contigs were also used as reference genomes for mapping VANA reads, to accurately reconstruct the complete genomes of circular DNA viruses, using the Geneious algorithm with the same parameters as before. Finally, the close-to-complete genomes of detected viruses were annotated using BLASTx [51] based on the NCBI protein (nr) database, the conserved domains database (https://www.ncbi.nlm.nih.gov/Structure/cdd/wrpsb.cgi/, accessed on 8 October 2022) for checking functional gene domains, and the HMMER web server (https://www.ebi.ac.uk/Tools/hmmer/search/phmmer/, accessed on 8 October 2022) for identifying conserved protein motifs. The open reading frames (ORFs) were also identified using the ORFfinder tool in Geneious Prime. Additionally, reads were classified using Kraken2 (ver 2.1.1) with default parameters, using the standard database (June 2020 release) containing records of k-mers and the lowest common ancestor (LCA) of all organisms. This process enabled the classification of reads and the obtaining of counts associated with the samples used as alien and internal positive controls within the Galaxy platform [52,53].

### 2.5. Confirmation of Detection by Polymerase Chain Reaction (PCR) and Sanger Sequencing

PCR primers and PCR/reverse transcription–polymerase chain reaction (RT-PCR) assays were designed to confirm the presence of viruses detected by HTS. Total nucleic acids were extracted from the pools of lyophilized leaves using the PureLink Viral RNA/DNA Mini Kit (Thermo Fisher Scientific, Belgium). After elution in 50 µL of RNase/DNase-free water, cDNA synthesis was conducted using random hexamers and SuperScript III (Invitrogen, USA), following the manufacturer’s instructions. PCRs were performed using Mango Taq polymerase (Bioline, Belgium) with the following thermocycling program conditions: initial denaturation at 94 °C for 60 s, followed by 40 cycles of 94 °C for 20 s, 60 °C for 30 s, and 72 °C for 30 s, and final extension at 72 °C for 180 s. Amplicons were visualized on 1% agarose gel stained with GelRed^®^ (Biotium, Fremont, CA, USA). PCR amplicons were purified using Nucleospin^®^ Gel and PCR clean-up (Macherey-Nagel, Düren, Germany). They were then Sanger-sequenced from both senses, to confirm sequence identity, by Macrogen Europe BV (Amsterdam, The Netherlands). Amplicon sequences were visualized, corrected, and trimmed in Geneious Prime (ver 2020.2). Final sequences were mapped to the target virus genome to confirm its identity.

### 2.6. Phylogenetic Analyses

To determine the taxonomic status of the newly discovered and previously reported species within the *Potyviridae*, *Solemoviridae*, *Geminiviridae*, and *Alphasatellitidae* families, sequence comparisons and phylogenetic analyses were conducted based on the amino acid sequences of the polyprotein for the Potyvirus group, the replicase protein for the Mastrevirus and Alphasatellite groups, and the nucleotide sequences of full-length genome sequences for BWYV of the Polerovirus group. The obtained sequences from this study and viruses from the same family were aligned using Geneious and then trimmed to ensure uniform sequence lengths. Subsequently, phylogenetic trees were constructed using the MEGA11 program, employing both neighbor-joining (NJ) and maximum likelihood (ML) methods with 1000 bootstrap values to assess the robustness of the tree topology [54]. The tree was visualized using the tool iTOL (v6.8) (https://itol.embl.de/, accessed on 12 May 2023) [55].

### 2.7. Identification of SNPs in Each Pooled Sample and In-Depth Population Analyses

For some virus species detected in the samples, a reference genome was selected, either from our own dataset for new viruses or from the NCBI refseq database (ver 1.1) for known viruses, based on the closest genome sequence from our isolates. The reads of each pooled sample were mapped against the reference genome of NCBI (for known viruses) with the parameter “Low sensitivity/Fastest” (the percentage of tolerated mismatches was applied based on the demarcation criteria described in Section 2.4). Then mapped reads were used to find SNPs in nucleotide alignment. SNP calling (Geneious Prime, ver 2020.2) was performed based on the default parameters (Minimum Coverage: 20; Minimum Variant Frequency: 0.2; Minimum Strand-Bias > 65%; *p*-value: 10^−5^). The results of single-nucleotide polymorphism (SNP) calling were used to study virus populations in the pooled samples as follows: R software version 4.0.3 (http://www.r-project.org/, accessed on 2 February 2023), including the ade4 and factoextra libraries, was used to conduct principal component analysis (PCA) based on the SNP table of each sequenced pool as previously described [56]. To evaluate the mean extent of genetic variation within a population of sequences, SNPGenie [57] was applied to the SNP table (SNP Reports, Geneious format) to calculate the mean gene diversity at all nucleotide sites in the viral genomes of each pooled sample.

### 2.8. Visualization of the Virome Network

ORA-LITE software (ver 3.0.9.142) (netanomics.com) was used to visualize the virome network in the saffron samples from the ten provinces, as previously described [35]. This visualization aimed to illustrate how virus species are distributed across provinces and to identify potential associations between virus taxa and saffron plants in these regions. A definition matrix was subsequently created, including sample codes from various provinces and the viruses detected in each sample (Table S2).

## 3. Results

### 3.1. Generation of HTS Data and Quality Control of the Sequencing

None of the libraries were flagged as of poor quality based on FASTQC analysis, with a mean quality value across the base positions exceeding 32. We obtained 32,678,514 forward reads and an equal number of reverse reads from 20 pooled samples of cultivated saffron *C. sativus*, along with two wild saffron pooled samples and external alien controls (EACs). The total number of “both” and “single” reads was 21,822,917. Details regarding the number of reads per pooled sample after demultiplexing can be found in Supplementary Table S3. The proportion of reads with quality scores above 30 (Q30) ranged from 91% to 99% across the samples (Table S4). The number of “both” and “single” reads passing quality control ranged from 258,248 to 1,610,020 per pooled sample. The RCA yielded high-quality reads (Q30) ranging from 96% to 99%, with a total read count of 34,710,030.

After read analysis using Kraken2, up to eight reads per pooled sample were assigned to the IPC (BBrMV). In ten pooled samples from two libraries, no read related to BBrMV was detected, while one to eight reads were found in the other twelve samples. On the other hand, reads of PvEV-1, -2, and -3 infecting the EAC were obtained in large numbers for Library 1 (16,309 reads over a total of 2,750,306 for Alien 1) but in low numbers for Library 2 (11 reads over a total of 2,240,278 for Alien 2) (Table S5). Additionally, no reads related to the three endornaviruses were observed in saffron samples from Library 2, while zero to five reads were detected in saffron samples from Library 1 (Table S5).

### 3.2. Contig Generation and Virus Identification

The average number of contigs obtained after de novo assemblies was 1214 contigs per sample. The minimum number of contigs was 93 in Sample H16 from Kerman province, while the maximum was 5649 in Sample H9 from Afghanistan (Table S4). The number of contigs generated from RCA was 175,554 contigs, and these contigs facilitated the completion and reconstruction of the ssDNA circular genome of viruses detected by VANA. In cases where genome coverage was low, RCA data provided additional sequences that ensured more-complete genome assembly of ssDNA viruses.

The detected agents (see Table 1) included viruses from three distinct orders—*Patatavirales* (*Potyvirus*), *Sobelivirales* (*Polerovirus*), and *Geplafuvirales* (*Mastrevirus*)—as well as a satellite DNA element from the family *Alphasatellitidae* (*Clecrusatellite*). A maximum of five virus species were detected in the samples from the Razavi Khorasan, Kerman, and Fars provinces. The lowest number of detected viruses was two for the Qom, Markazi, and Semnan provinces. Saffron latent virus (SaLV) was ubiquitous and found in all samples from cultivated saffron but not in the two wild saffron samples. Additionally, wheat dwarf virus-associated alphasatellite (WDVaA) was detected in samples from half of the provinces that were sampled, while saffron dwarf virus was also present in the same samples (H03, H04, H06, H09, H11, H12, H14, H17, H18, H19). A putative new *Potyvirus* species, characterized hereunder, was specifically detected in the Razavi Khorasan, Fars, and Tehran provinces. Wheat dwarf virus and beet western yellows virus were exclusively detected in the Fars and Kerman provinces, respectively.

The sample from Afghanistan contained a diverse array of known viruses, including SaLV, SYMV, SaDV, and WDVaA. Moreover, for the putative new polerovirus identified in Afghanistan, four contigs (PV831655, PV831656, PV831657, and PV831658), with sizes ranging from 396 to 628 bp, presented homologies with various *Polerovirus* species with BLASTx identity scores spanning from 53% (P1-P2 protein) to 79% (P2) with the nr database. The contigs were not overlapping and covered 2032 nucleotides, representing close to half of the complete *Polerovirus* genome.

In wild saffron (H22, *C. speciosus*), turnip mosaic virus (TuMV) was the sole detected virus. The reconstructed TuMV genome spans approximately 9405 nucleotides, nt, and shares 98% identity with the isolate IRN TRa6 (AB440238), identified in Iran in bastard cabbage (*Rapistrum rugosum*) [58]. This is the first virus known to infect *C. speciosus*. No virus was detected from the second wild saffron species, *C. caspius*.

After generating the contigs, the genomes of the identified viruses were reconstructed, followed by ORF identification. The consensus genome sequences of all identified viruses (BWYV, SaIRV, SaDV, SaLV, SYMV, TuMV, WDV, and WDVaA) in the different samples were deposited in GenBank (Table S6).

### 3.3. Confirmation of Detection by PCR and RT-PCR

To strengthen the robustness of the HTS detection, we conducted further confirmation using PCR and RT-PCR, completed by Sanger sequencing of PCR products. This validation was performed on BWYV, SaIRV, SaDV, SaLV, SYMV, TuMV, WDV, and WDVaA. All PCR and RT-PCR detections were successful using the designed primer pairs, with sizes matching the expected values (as shown in Figure S3). The sequences obtained from Sanger sequencing were 93% to 100% identical to the sequences obtained by HTS data. These sequences maintained the same organization in terms of nucleotide sequence and ORFs (Table S7) and have been submitted to NCBI under the following accession numbers: BWYV, PV831727; SaIRV, PV791990; SaDV, PV831724; SaLV, PV791991; SYMV, PV791989; TuMV, PV831726; WDV, PV831725; and WDVaA, PV829650.

### 3.4. Field Surveys and Virus Detection

In total, seven virus species and one alphasatellite were detected in the analyzed samples, including seven known species (SaLV, SYMV, TuMV, SaDV, WDV, BWYV, and WDVaA) and one putative new species belonging to the Potyviridae family, tentatively named SaIRV (saffron Iran virus) or *Potyvirus crociranense*. Among the eleven sampled provinces, more than one sample was collected from five provinces: Kerman, Razavi Khorasan, Semnan, South Khorasan, and Yazd. The virus detection across different samples within each province was mostly consistent. Specifically, in Kerman province, three samples were taken where SaLV, SYMV, and SaDV were detected in all samples, while WDVaA was present in two samples, and TuMV and BWYV were detected in only one sample. In Razavi Khorasan, SaLV, SYMV, and SaDV were present in all samples, and SaIRV and TuMV were found in two samples. Additionally, WDVaA was detected in one sample. Two samples come from Semnan province, both containing SaLV and SaDV, but only one sample had SYMV. In South Khorasan, SaLV, SYMV, and SaDV were present in all samples, while WDVaA was found in two of them, and TuMV in only one sample. Finally, the two samples of Yazd province were both infected by SaLV, SYMV, SaDV, and WDVaA.

There was no clear correlation between the surface dedicated to saffron cultivation and the presence of viruses (Figure S2). Yet, the high difference in cultivation area between Razavi Khorasan and other provinces makes direct comparisons challenging. In Razavi Khorasan, which has the largest cultivation area, almost all viruses except WDV and BWYV were detected in the pools.

### 3.5. Virome Network

We hypothesized that the distribution of virus species across provinces was associated with specific virus taxa and their interactions with saffron plants in different regions. To illustrate these potential associations, we employed network visualization. Different network layouts revealed that SaLV, SYMV, and SaDV were widespread. In the province view (Figure S9a), the positions of samples from different provinces remained fixed, and the arrangement of viruses helped us understand the network. Instead of clustering together, samples related to a single province were scattered across different places. In the virus view (Figure S9b), the viruses were positioned at the center of the network, and the samples from different provinces were organized around them. Both layouts revealed a mix of viruses in each sample.

**Figure 2:**
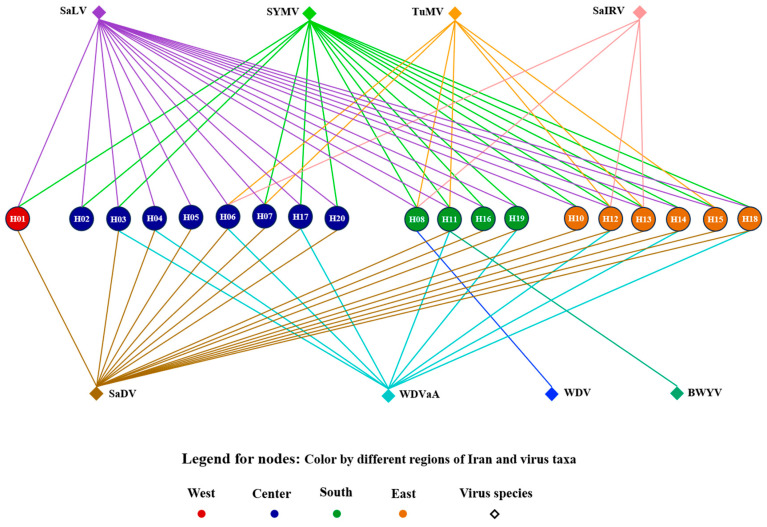
Hierarchy Layout of the virome network for virus species detected in saffron samples from various regions of Iran.

In the Hierarchy Layout of the virome network, samples related to each province are kept in the middle of the figure, with different colors distinguishing them based on the west, center, south, and east of Iran. All potyviruses are at the top of the figure, while others (including SaDV, WDVaA, WDV, and BWYV) are below. This layout clearly organizes detected viruses in each part of Iran. There is no specific region associated with a particular virus; instead, each virus is detectable from different geographical origins (except WDV and BWYV, which were detected in just one sample). In conclusion, the visualization suggested the presence of a mix of viruses distributed without geographical clustering (Figure 2).

### 3.6. Genome Reconstruction of Known Viruses and Alphasatellite Identified in Saffron

Three potyviruses were detected and their genomes sequenced. First, *Potyvirus crociranense* (saffron latent virus—SaLV) was detected in 20 samples across various provinces. Twenty nearly complete genomes were obtained, sharing between 97% to 99% nucleotide (nt) sequence identity with each other. The range of sequence lengths was 9119–9550 nt. The highest homology with publicly available genomes was 98% with SaLV isolate Ir-Kh1 from saffron in Iran (KY562565, [59]).

Saffron yellow mosaic virus (SYMV) was detected in most samples (n = 17), except for H04 (Markazi), H05 (Semnan), and H06 (Tehran). Fifteen nearly complete genomes were reconstructed, exhibiting 89–99% nt sequence identity with each other, and their lengths ranged from 9410 to 9640 nt. Additionally, two incomplete genomes were obtained from samples H2 and H7, with the polyprotein ORF not completely sequenced, and presented 8931 and 9326 nt lengths. In the ML phylogenetic tree (Figure S4) constructed from the polyprotein sequences, the SYMV from the Afghan sample was an outlier in comparison to the other samples from Iran. Finally, *Potyvirus rapae* (turnip mosaic virus—TuMV) was detected in seven samples from the Tehran, Isfahan, Fars, Kerman, and Khorasan provinces, as well as one sample from wild saffron (*C. speciosus*, H22). The five nearly complete TuMV sequences shared 84–99% nt sequence identity with each other, and their lengths ranged from 9644 to 9804 nt. Additionally, three incomplete genomes presented lengths ranging from 9186 to 9569 nt. The highest homology with publicly available genomes was 98% with the isolate IR-SKH genome sequence from saffron in Iran (OK632023, [18]). In the phylogenetic tree with complete genomes, two sequences (PV831704, PV831705) clustered together in one clade with an isolate from saffron in Iran (WKU78709), sharing an identity of 84–85% with other sequences. The three other sequences (PV831706 from Fars, PV831707 and PV831708 from Razavi Khorasan) clustered in another distinct clade (Figure S5). The genetic sequence of all these potyviruses displayed characteristic features typically found in the *Potyvirus* genus. The large ORF encodes a polyprotein consisting of nine highly conserved potyvirus proteolytic cleavage sites and ten putative mature proteins: P1, HC-Pro, P3, 6K1, CI, 6K2, VPg, NIa, NIb, and CP.

The polerovirus BWYV (beet western yellows virus) was detected in H11 from Kerman province, with a nearly complete genome reconstructed (5597 nt) and 97% nt sequence identity with the genome of isolate IV 400 from citrus in the USA (MZ330108, [60]). Additionally, a phylogenetic tree based on the full-length nucleotide sequences confirms that BWYV was most closely related to BWYV isolate IV 400 (MZ330108, [60]) (Figure S6).

The mastrevirus SaDV (saffron dwarf virus) was present in all provinces except Qom and Fars. Among the 18 samples in which this virus was detected, six complete genomes could be generated with nt sequence identities ranging between 95 and 97% of each other and a genome size of 2726 nt. There were also twelve incomplete genome sequences with a size range of 2084–2644 nt. The highest genome identity was 97% with an isolate from India (BK067261, [20]). Like other mastreviruses, the genome sequence comprised four ORFs that encode movement protein, coat protein, Rep A and Rep proteins, and the origin of replication (5′–TAATATTAC–3′) within a stem-loop structure. A phylogenetic tree, based on the ML method using two full-length genomes and replicase proteins (RepA), clearly showed that the sequence generated in our study closely aligns with the genome of SaDV (Figure S7). Pairwise identity analyses using clustalW revealed that the Rep gene of our sample shows 96.2% identity with the sequence from Iran (PQ3. 192009) and 96.6% identity with the sequence from India (BK067261).

A second mastrevirus (wheat dwarf virus—WDV) was specifically detected in Fars province, with an incomplete genome of 2538 nt. This WDV isolate shares 98% nt sequence identity with isolate CRNTA01 from *Triticum* sp. in Iran (KU877917, [61]). A phylogenetic tree, constructed using the ML method based on replicase proteins, clearly demonstrates that our study’s sequence clusters within the same clade as the WDV isolate from barley in Turkey (AJ783960, [62]) (Figure S7).

The wheat dwarf virus-associated alphasatellite (WDVaA) was detected in ten samples from the provinces of Kerman, Markazi, Razavi Khorasan, South Khorasan, Tehran, and Yazd. Among the ten sequences, two complete genome sequences, sharing 92% nt sequence identity with each other, were retrieved. Additionally, there were six incomplete sequences ranging from 1181 to 1413 nt. The genome pairwise comparison showed that the detected alphasatellite shared the highest identity of 89% with the WDVaA isolate VC95 from winter barley in France (PP445014, [63]), above the species demarcation criterion of 88% [64]. A phylogenetic tree of this alphasatellite, based on the ML method and Rep amino acid sequences, is shown in Figure S8. The alphasatellite groups with WDVaA, indicating a shared common ancestor within the genus *Clecrusatellite*. Furthermore, the nucleotide sequence tree of the complete genome delivered the same result.

### 3.7. Genomic Characterization of the Newly Discovered Virus

**Figure 3:**
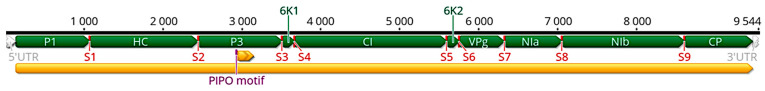
Genome organization of the new potyvirus. The orange arrow represents the viral genome’s open reading frames (ORFs). Green arrows indicate the specific proteins coded for. The labels S1-S9 correspond to predicted proteinase cleavage sites. The genome contains a single large open reading frame (ORF) encoding a polyprotein that is cleaved into 10 mature proteins: protein 1 (P1), the helper-component protease (HC-Pro), protein 3 (P3), 6 kDa peptide 1 (6K1), the cylindrical inclusion protein (CI), 6 kDa peptide 2 (6K2), the genome-linked viral protein (VPg), the nuclear inclusion-a protease (NIa-Pro), the nuclear inclusion-b protein (NIb), the capsid protein (CP), and a small frameshift-derived peptide (PIPO) from the P3 region.

**Figure 4:**
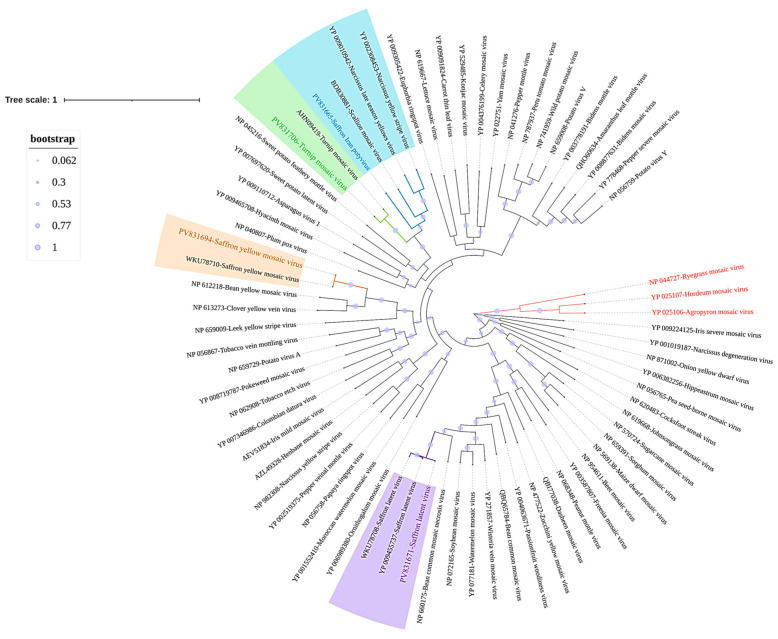
Maximum likelihood phylogenetic tree based on a multiple-amino-acid alignment of Potyvirus polyprotein sequences (using the Jones–Thornton (JTT) model with 1000 bootstraps). The tree includes potyviruses discovered in this study (SaIRV, SaLV, SYMV, and TuMV) and other members of the *Potyvirus* genus. Three *Rymovirus* species were used as outgroups (in red). Viral sequences identified in this study are highlighted in color. Circles at the nodes indicate bootstrap support, as explained in the adjacent legend. The analysis involved 70 amino acid sequences, with a total of 3864 positions in the final dataset. Evolutionary analyses were conducted using MEGA11.

Based on the demarcation threshold set by the International Committee on Taxonomy of Viruses (ICTV), members of different species within the *Potyvirus* genus, possessing complete ORF sequences, are generally considered distinct if they exhibit less than 76% identity in nucleotide sequence and less than 82% identity in amino acid sequence [65]. Our study identified a putative novel virus species with a maximum of 68% nucleotide similarity to another potyvirus, TuMV isolate GRC33 from *Brassica* sp. in Greece (AP017836, [66]), and 63% amino acid similarity to Narcissus yellow stripe virus (NYSV) isolate NY-KM1P from *Narcissus* in Japan (BBE01234, [67]). This newly discovered virus has been tentatively named *Potyvirus crociranense* (saffron Iran virus—SaIRV). It was detected in four samples from three provinces: Tehran, Fars, and Razavi Khorasan. The three nearly complete genomes shared 95–96% nt sequence identity with each other with lengths of 9544 to 9548 nt. A partial genome, containing a gap of 465 nt, has also been reconstructed. The proposed reference genome sequence (H08) of *SaIRv* (PQ740911) is 9548 nt long and includes a 5′-UTR of 136 nt and a 3′-UTR of 73 nt. Both UTRs are incomplete. The sequence contains a large ORF (nucleotides 137 to 9475) encoding a polyprotein of 3113 amino acid (aa) residues with a calculated molecular mass of 325.500 kDa. Notably, the sequence aligns with the typical characteristics of the *Potyvirus* genus, featuring a complete ORF. Nine highly conserved potyvirus proteolytic cleavage sites were found in the polyprotein (Figure 3), and these sites give rise to ten putative mature proteins: P1 (314 aa), HC-Pro (458 aa), P3 (355 aa), 6K1 (52 aa), CI (643 aa), 6K2 (53 aa), VPg (190 aa), NIa (243 aa), NIb (518 aa), and CP (286 aa). Highly conserved potyviral motifs were identified in the new potyvirus polyprotein, including a ^624^PTK^626^ motif in HC-Pro; ^1265^GAVGSGKST^1273^, ^1483^VATNIIENGVTL^1494^, ^1527^GERIQRLGRVGR^1538^, and ^1354^DECH^1357^ motifs in CI; and ^2615^GNNSGQPSTVVDNT^2628^ and ^2659^GDD^2661^ motifs in Nib [68]. The highly conserved motif ^2932^GAAAAAAA^2938^ and a small ORF (PIPO—Pretty Interesting *Potyviridae* ORF) encoding 70 aa were detected in the P3 protein, and the calculated weight of the PIPO is 7.96 kDa. Evolutionary relationships were investigated alongside complete TuMV, SYMV, SaLV, and SaIRV polyprotein on an ML tree. The known potyviruses (TuMV, SYMV, and SaLV) were grouped with their corresponding virus isolates, validating the expected evolutionary relationships. The phylogenetic tree indicated that the newly detected potyvirus from saffron grouped with scallion mosaic virus (BDB30886), Narcissus late season yellows virus (YP_009010942), and Narcissus yellow stripe virus (YP_002308453). Along with genome structure and similarity, phylogenetic analyses further support that the newly characterized *Potyvirus* from saffron represents a novel species (Figure 4).

### 3.8. Genome-Wide Analyses of Virus Diversity

Our hypothesis posits significant variability in virus genomes within each sample, and geographical clustering of virus composition across different provinces. We conducted an analysis of SNP patterns and compared consensus sequences for SaLV (20 sequences), SYMV (17 sequences), TuMV (8 sequences), SaDV (18 sequences), and WDVaA (10 sequences) to explore population diversity within and between sampled provinces in Iran. Nearly complete sequences were used for SYMV and SaLV. However, for SaDV, WDVaA, and TuMV, only portions of the genome present in multiple samples were utilized, as the sequences were not complete in all samples.

A total of 2652 SNPs (21% of the genome) were identified with a frequency between 20 and 96% using reads mapped to the closest SaLV reference genome from Iran (NC_036802). Among the 20 samples analyzed, 188 SNPs were found in over 60% of the samples. Notably, 86 (46%) of these SNPs were associated with the 5′ part of the genome (P1, HC, and P3). In total, 2663 SNPs were identified by mapping alignment reads (from 17 samples) against the average sequenced genome of SYMV (H13). Of these, 18 SNPs were found in over 60% of the samples, and 40 SNPs were specifically associated with the CI gene. For TuMV, 154 SNPs were detected at CP positions (9% of the polyprotein CDS) by aligning used reads for mapping (from seven samples) against the closest TuMV reference CP gene from Iran (AP017791), and 17 of these SNPs were found in over 60% of the samples. For SaDV, 222 SNPs were identified by aligning the reads from 13 samples against the average sequence of the SaDV Rep gene (H7), which spans 966 nt, and 21 SNPs were found in over 60% of the samples. For WDVaA, 164 SNPs were found in the ten samples for the Rep gene and 14 SNPs were found in over 60% of the samples (Supplementary Tables S8–S12).

**Figure 5:**
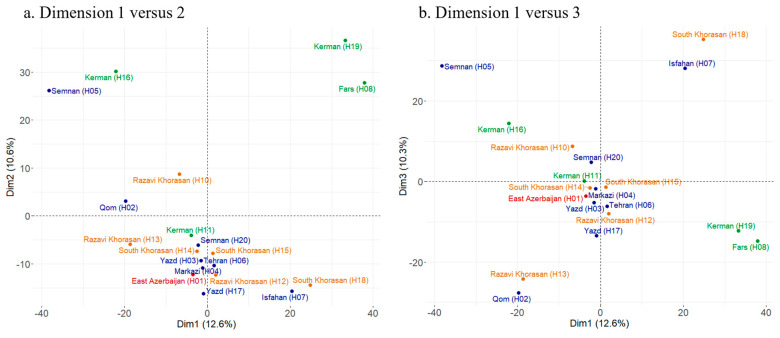
Principal component analysis (PCA) of saffron latent virus (SaLV) populations reveals the first, second, and third dimensions obtained using all the detected single-nucleotide polymorphisms (SNPs) as variables for SaLV sequences from 10 provinces. (**a**). PCA—Dimension 1 versus 2. (**b**). PCA—Dimension 1 versus 3. The colors represent different regions of Iran: red (west), blue (center), green (south), and orange (east).

Furthermore, PCA was conducted on SNP frequencies for each species to explore any geographical linkage among detected viruses. In the PCA, no discernible geographical clustering of diversity was observed whatever the viral species. For instance, in the case of SaLV (Figure 5a,b), most samples clustered around the center in the PCA plot, indicating that they were not clustered by the principal components (PCs). However, a few samples formed a distinct group. Notably, Sample H2 from Qom and H13—the third sample from Razavi Khorasan—were grouped. This observation is intriguing since Qom is centrally located in Iran, while Razavi Khorasan lies in the east. Additionally, other samples from Razavi Khorasan (H10 and H12) were positioned at the center of the PCA plot. Conversely, samples from Kerman (H11, H16, H19) were scattered across different locations and did not form a cohesive group. The estimated gene diversity across all nucleotide sites in the genome of the pooled sample ranged from 0.023 to 0.039, which supported the absence of clustering observed in the PCA (Table S13).

## 4. Discussion

Sensitivity and controls. It has been demonstrated recently that HTS-based virus detection can be reliably used in surveillance and diagnostic programs, and its use was leveraged by decreased sequencing costs [41,69,70]. Additionally, utilizing HTS within these surveillance systems showed enhancement in the ability to promptly tackle disease threats, thus supporting effective management strategies [71]. The VANA approach has demonstrated high efficiency in virus discovery and etiology studies [45,72,73]. One of the key advantages of the VANA approach is that it allows for the pooling of plant samples while maintaining appropriate analytical sensitivity, enabling the study of a larger number of specimens from diverse locations, as already demonstrated in previous studies [41,45,74]. A comprehensive review revealed that the VANA approach detected numerous viruses from 85 different families between 2010 and 2020, underscoring its robust capability to identify both DNA and RNA viruses across various viral families [41]. Furthermore, the VANA approach is capable of detecting capsidless viruses, such as those in the Endornaviridae family. However, this technique has been reported to exhibit lower efficiency in detecting viruses with low titers and less stable particles [41,75,76]. Another study indicated that while the dsRNA-based approach offers a more-complete representation of the RNA virome, especially for high-complexity ones, the VANA approach was a reasonable alternative for low- to medium-complexity viromes [74]. This aligns with our study, particularly for the analysis of DNA viruses. Among the viruses detected in this study using VANA, the presence of both SaDV and WDVaA was confirmed using the RCA data, reinforcing these findings and ensuring accurate genome reconstruction of SaDV and the WDVaA.

As contamination is common in different steps of the molecular biology procedure associated with HTS-based virus detection, it has been suggested to use control samples from extraction to sequencing to identify contamination [40,49]. Based on international guidelines for pest detection by HTS [44], it is recommended to use an alien control to monitor contamination throughout the HTS protocol. The IPC, a reference banana sample, exhibited a very low concentration for the alien virus (BBrMV). This low concentration, combined with the pooling of 50 samples, may explain the limited detection sensitivity. Additionally, it underlined the detection limit of our protocol: a low-abundance virus present in a single sample could be missed by the pooling approach. In contrast, another study found that a relatively high number of reads mapped to the internal alien virus species could limit the sensitivity for detecting viruses in the analyzed samples [77] (in revision). This variability is reinforcing the need to have a stable, lyophilized, and homogeneous alien control, but this might not be enough when using pooled samples to stabilize the proportion of IPC reads, as the proportion will depend on the viral concentration in the analyzed samples. Nonetheless, the situation of a low number of IPC reads encountered in this publication might be preferable to a high number of reads of the internal alien viruses, as the latter would limit the sensitivity of virus detection. For the external control, it should be noted that the alien control of Library 1 was detected efficiently (16,309 reads based on direct read classification), while that of Library 2 was poorly detected. Nevertheless, cross-contamination between samples could be evaluated with Alien 1 and was very low (maximum of five alien reads in any other sample). Our threshold for considering a virus detection event the result of cross-contamination was therefore set at 0.03% (5/16,309) of the total reads from the sample with the highest abundance of a virus (Table S5). The saffron viruses were not detected in the external alien controls. Overall, these results indicate a very low level of cross-contamination between samples. Additionally, (RT-)PCR tests confirmed the presence or absence determined by HTS of all tested viruses in the different samples.

After generating the data, various bioinformatic tools, such as Kraken2, Galaxy, and Geneious, were used on the generated datasets. Each tool had its own strengths and limitations, so they could complement each other to strengthen the results. Applying multiple bioinformatic approaches in parallel can help validate findings [40] and improve the accuracy of annotations [78], and should therefore be recommended.

Virome. The ICTV demarcation criteria were used to evaluate whether the detected viral contigs belonged to known or new species [79]. Unlike other viral sequences from various organisms and samples, plant viruses are most probably under-represented in databases [39]. However, studies like ours contribute to expanding this type of data in the international nucleotide sequence database. This study contributed to the submission of 83 complete or nearly complete nucleotide sequences to NCBI, among which are 4 genomes of new potyvirus species and 79 genomes of known species (Table S6). Our results significantly enrich the NCBI database, which previously only included about 100 partial and complete nucleotide sequences of viruses infecting saffron plants. These genomes were obtained from pooled saffron plant samples, as in many HTS studies, where samples are pooled together rather than processed individually during surveys [80,81,82]. However, this approach carries the potential risk that the consensus genome may be a chimera of different isolates infecting different samples in the pool. This phenomenon can also occur within a single sample if several isolates are present. This risk was addressed in several ways during our study: comparing consensus sequences obtained by de novo assembly and read mapping, reviewing alignments, observing SNP frequencies, comparing the genomes obtained between samples, and careful validation of the ORFs, motifs, and conserved sequences. We were previously able to reconstruct Ugandan cassava brown streak virus (UCBSV) genomes up to the haplotype level on pooled samples [56]. Although this sample pooling method carries certain risks, it offers the advantage of increasing read mapping in pooled sequencing compared to individual sequencing [83].

Based on the results, TuMV was the only plant virus which was detected in *C. speciosus*, a wild saffron species. According to a recent study, three wild saffron species are present in the north of Iran: *C. caspius*, *C. speciosus*, and *C. almehensis* [84]. During a recent survey of SaLV prevalence in these species, positive samples were only detected for *C. almehensis* [85]. In this study, the sampling of wild species was performed at the experimental farm of the University of Tehran, established one kilometer away from cultivated saffron (*C. sativus*) sites. On average, a greater richness of virus species (3.9 per pool) was detected in cultivated saffron (*C. sativus*) compared to wild saffron (0.5 per pool). Yet, more sampling is needed to gain a better understanding of potential exchanges between wild and cultivated saffron, as conducted for cucurbitaceae in Spain [86] and Apiaceae in France [87].

Virus detection and prevalence. SaLV, TuMV, SYMV, and SaDV had been previously reported in Iran [18,21,59,88]. However, WDV and WDVaA are novel findings for Iranian saffron. The prevalence of different virus species among sampled provinces includes SaLV at 100%, SYMV and SaDV at 82%, TuMV at 45%, SaIRV at 27%, BWYV and WDV at 9%, and WDVaA at 55%. Overall, these results indicate that viruses are widespread across Iran’s saffron-producing regions. This widespread presence could be attributed to vegetative propagation, as noted in another study about the prevalence of SaLV [59]. The discovery and characterization of saffron Iran virus (SaIRV) adds one more potyvirus species infecting saffron. So far, potyviruses can be considered the most diverse and prevalent viruses infecting saffron worldwide. Additionally, recent studies showed widespread presence of viruses in saffron from different countries such as China, Hungary, India, Italy, and Spain [19,20]. So far, the impacts of these viruses on the quality and yield of saffron have been poorly studied except for SaLV [24,25] and CMV [26].

In our study, there was no clear correlation between the cultivation surface in a province and the number of detected viruses. It is important to note that other factors, such as local agricultural practices, climate conditions, and virus transmission mechanisms, might play significant roles in virus prevalence [75,89,90]. In addition, the virome network did not show any trends in the presence of viruses among provinces. Importantly, the experimental design of this study aimed to identify the viruses present in each province, not their prevalence at the province or field level nor their impact on yield. Our results provide a baseline of viruses circulating in saffron fields in Iran and could be further complemented by characterizing their impact on yield and quality. For example, the recently published biological characterization framework [91] could be followed to biologically characterize one or several viruses (SYMV and the new potyvirus species as priorities), with emphasis given to greenhouse inoculation experiments and traditional (RT-)PCR-based targeted prevalence studies on symptomatic and asymptomatic samples analyzed individually. Further research should also focus on a complementary survey of the virus species that currently have a distribution restricted to some provinces in Iran, such as BWYV and WDV.

The only virus that had been previously reported from Afghanistan is SaLV [92]. To the best of our knowledge, our study marks the first report of SYMV, SaDV, and WDVaA in Afghanistan. Our study also identified four contigs from Afghanistan with homologies to Polerovirus species. The homologies were low, with maximum homologies of each contig ranging from 53% to 79%, suggesting putative new species, whose characterization would require further studies. On the other hand, close nucleotide pairwise identity (>95%) was observed between virus sequences of Afghanistan and the provinces of Iran, with the exception of SYMV with a maximum identity of 90.4% with Iranian genome sequences and a clear separation in the phylogenetic tree (Figure S4).

To refine the analysis of genetic diversity within and among provinces, the presence and pattern of minor SNPs (<50%) were studied at the genome or gene level for SaLV, SYMV, TuMV, SaDV, and WDVaA. High genetic diversity was observed within each pooled sample, but there was no differentiation between the provinces (no clear geographical clustering) from PCAs. These results are consistent for the studied virus species. So, while the genetic variability was high, it was equally spread across the regions, regardless of the virus. This observation aligned with the results from the virome network of the absence of a geographical pattern for virus presence, emphasizing the overall genomic homogeneity across different regions without clear geographical clustering between provinces. In contrast, a similar methodology identified three distinct haplotypes of the Uganda cassava brown streak virus with clear geographical clustering at the national scale in Rwanda [56].

This absence of clustering might originate from the phytotechnical management of saffron. As saffron is vegetatively propagated, the planting material will be infected by the systemic viruses infecting the mother plant [93]. Saffron was initially mainly grown in the eastern provinces. Based on a statistical report from the Iranian Ministry of Agriculture, for more than 15 years, its cultivation has extended to the rest of the country to answer the growing demand of saffron. Sadly, the absence of knowledge on virus presence and impact on yield precluded the setup of phytosanitary certification schemes. Consequently, it is likely that the viruses present in the main corms from Eastern Iran have been transferred to other provinces and spread throughout the country via vegetative propagation. Additionally, a study by Alavi-Siney et al. [94] on saffron plants found that genetic differentiation among saffron ecotypes increases with geographic distance. However, ecotypes from seven provinces in Iran were grouped into two major clusters without geographical clustering. Nevertheless, the complexity of the relationship between viral and saffron plant diversity needs to be further studied side by side on the same plants to capture the biological reality.

Most pest management methods in Iran are traditional and need to be strengthened using new technologies [95]. As part of a robust management scheme, it is advised to prevent the dissemination of viruses by planting only certified saffron material, keeping healthy breeding material [19] and testing for viruses through laboratory tests. This testing should focus on the most damaging viruses affecting saffron. However, more biological data is needed to refine virus selection for certification programs. Given the broad genetic variability and adaptability of potyviruses, as well as their economic significance [96,97], SYMV might be the top priority for biological characterization and, if it poses a phytosanitary risk, inclusion in certification testing. SaIRV, the newly identified Potyvirus present in only three provinces, is another priority given its currently restricted dissemination and the absence of biological information. The virus species currently detected in only some provinces (such as BWYV and WDV) should also be considered a priority to limit their potential geographical expansion.

## 5. Conclusions

Combining VANA-HTS virome detection and targeted (RT-)PCR-based tests, our research has described both known and novel virus species infecting saffron on a national scale in Iran, the world’s largest saffron producer. No geographical clustering of virus species or haplotype presence was observed. This newly acquired knowledge of virus presence and diversity is crucial for further evaluation of risk. Should significant risks be identified, it is recommended to study propagation systems more closely, particularly by testing the corms of saffron, to minimize the spread of high-risk viruses.

These advancements are vital for developing targeted strategies to manage and mitigate the potential impacts of viruses on saffron crops and pave the way for consistent protection of its economically and socially important cultivation.

## Figures and Tables

**Table 1:** Total number of reads generated in each pooled sample and assigned to the virus species detected in saffron samples.

Sample	Province	BWYV	SaDV	SaIRV	SaLV	SYMV	TuMV	WDV	WDVaA
H01	East Azerbaijan		1089		3603	41,434			
H08	Fars			10,880	32,298	54,888	11,244	2646	
H11	Kerman	829	1811		164,841	16,382	2121		626
H16			632		22,497	138,300			
H19			3080		24,058	99,195			586
H10	Razavi Khorasan		14,216		56,464	556,476			
H12			1220	8738	29,611	88,026	5291		1288
H13			2522	3828	101,407	243,496	4210		
H14	South Khorasan		370		31,722	111,194			1383
H15			1419		14,725	119,088			
H18			2270		20,086	283,666	5660		4340
H07	Isfahan		1661		30,914	69,878	2367		
H04	Markazi		2088		39,189				296
H02	Qom				7459	101,737			
H05	Semnan		546		96,586				
H20			3036		115,447	116,671			
H06	Tehran		4796	2024	50,412		33,469		8676
H03	Yazd		201		3842	102,213			608
H17			4632		98,318	299,031			1187
H09	Afghanistan		4782		44,518	11,586			927

## Data Availability

The data presented in this study are openly available in the NCBI Sequence Read Archive (SRA) under BioProject accession number PRJNA1187297, accessible at https://www.ncbi.nlm.nih.gov/bioproject/PRJNA1187297 (accessed on 4 February 2025).

## Supplementary Materials

The following supporting information can be downloaded at https://www.mdpi.com/article/10.3390/v17081079/s1, Figure S1: Symptoms of some collected plants. Figure S2: Table and scatter plot illustrating the relationship between the cultivation area (*x*-axis) and the number of detected viruses (*y*-axis). Figure S3: Electrophoresis Gel of PCR and RT-PCR products using the designed primer pairs to confirm high-throughput sequencing (HTS) results. Figure S4: Maximum likelihood phylogenetic tree based on a multiple-amino-acid alignment of SYMV polyprotein sequences (using the Jones–Taylor–Thornton (JTT) model with 1000 bootstraps). Figure S5: Maximum likelihood phylogenetic tree based on a multiple-amino-acid alignment of TuMV polyprotein sequences (using JTT model with 1000 bootstraps. Figure S6: Maximum likelihood phylogenetic tree based on a multiple-nucleotide alignment of BWYV full-length genome sequences (using the Tamura–Nei model with 1000 bootstraps). Figure S7: Maximum likelihood phylogenetic tree based on a multiple-amino-acid alignment of Mastrevirus replicase protein sequences (using the Le_Gascuel_2008 model with 1000 bootstraps). Figure S8: Maximum likelihood phylogenetic tree based on a multiple-amino-acid alignment of Alphasatellite replicase protein sequences (using JTT model with 1000 bootstraps). Figure S9: Visualization of the virome network for the virus species detected in saffron samples from various provinces of Iran. Table S1: Saffron samples collected from different provinces. Table S2: Virus-samples matrix used in the virome network. Table S3: The number of reads after demultiplexing. Table S4: Quality and number of reads and contigs in each pooled sample. Table S5: Kraken results for internal and external control reads. Table S6: GenBank accession numbers of identified viruses. Table S7: PCR and RT-PCR Based HTS Verification. Table S8: A complete list of SNPs in SaLV populations. Table S9: A complete list of SNPs in SYMV populations. Table S10: A complete list of SNPs in TuMV CP gene populations. Table S11: A complete list of SNPs in SaDV Rep gene populations. Table S12: A complete list of SNPs in WDVaA Rep gene populations. Table S13: Mean gene diversity estimated using SNPGenie.

## Abbreviations

The following abbreviations are used in this manuscript:

EAC	External Alien Control
HTS	High-Throughput Sequencing
IPC	Internal Positive Control
RCA	Rolling Circle Amplification
SaIRV	Saffron Iran Virus
VANA	Virion-Associated Nucleic Acids

## References

[1] Hill T. (2004). The Contemporary Encyclopedia of Herbs & Spices: Seasonings for the Global Kitchen.

[2] Taheri-Dehkordi A., Naderi R., Martinelli F., Salami S.A. (2020). A robust workflow for indirect somatic embryogenesis and cormlet production in saffron (*Crocus sativus* L.) and its wild allies; *C. caspius* and *C. speciosus*. Heliyon.

[3] Lopez-Corcoles H., Brasa-Ramos A., Montero-Garcia F., Romero-Valverde M., Montero-Riquelme F. (2015). Phenological growth stages of saffron plant (*Crocus sativus* L.) according to the BBCH Scale. Span. J. Agric. Res..

[4] Shahnoushi N., Abolhassani L., Kavakebi V., Reed M., Saghaian S. (2020). Economic analysis of saffron production. Saffron.

[5] Salehi A., Shariatifar N., Pirhadi M., Zeinali T. (2022). An overview on different detection methods of saffron (*Crocus sativus* L.) adulterants. J. Food Meas. Charact..

[6] Yousefi M., Shafaghi K. (2020). Saffron in Persian traditional medicine. Saffron.

[7] Fekrat H. (2003). The application of crocin and saffron ethanol-extractable components in formulation of health care and beauty care products. I International Symposium on Saffron Biology and Biotechnology 650.

[8] Kyriakoudi A., Ordoudi S., Roldán-Medina M., Tsimidou M. (2015). Saffron, a functional spice. Austin J. Nutr. Food Sci..

[9] Jafari S.-M., Tsimidou M.Z., Rajabi H., Kyriakoudi A. (2020). Bioactive ingredients of saffron: Extraction, analysis, applications. Saffron.

[10] Vahedi M., Kabiri M., Salami S.A., Rezadoost H., Mirzaie M., Kanani M.R. (2018). Quantitative HPLC-based metabolomics of some Iranian saffron (*Crocus sativus* L.) accessions. Ind. Crops Prod..

[11] Ahrazem O., Rubio-Moraga A., Castillo-López R., Trapero-Mozos A., Gómez-Gómez L. (2010). Crocus sativus pathogens and defence responses. Functional Plant Science and Biotechnology.

[12] Russo M., Martelli G., Cresti M., Ciampolini F. (1979). Bean yellow mosaic virus in saffron/il virus del mosaico giallo del fagiolo in zafferano. Phytopathol. Mediterr..

[13] Chen J. (2000). Occurrence and control of mosaic disease [turnip mosaic virus] in saffron (*Crocus sativus*). Zhejiang Nongye Kexue.

[14] Liao F.R., Lin W.Z., Chen X.H., Chen Q., Chen H.Y., Huang P.Y., Fang Z.P., Wu Y., Shen J.G., Lin S.M. (2017). Molecular identification and sequence analysis of Ornithogalum mosaic virus in saffron (*Crocus sativus*) corms. Sci. Agric. Sin..

[15] Parizad S., Dizadji A., Koohi Habibi M., Winter S., Kalantari S., Movi S., García-Arenal F., Ayllón M.A. (2018). Description and genetic variation of a distinct species of Potyvirus infecting saffron (*Crocus sativus* L.) plants in major production regions in Iran. Ann. Appl. Biol..

[16] Zheng H., Wu X., Han K., Chen Z., Song X., Peng J., Lu Y., Lin L., Chen J., Yan F. (2018). First Report of Beet western yellows virus Infecting *Crocus sativus* in China. Plant Dis..

[17] Tavoosi M. (2022). Molecular Detection and Investigation of Irish severe mosaic virus in Saffron, Fields of Razavi and South Khorasan. J. Saffron Res..

[18] Tavoosi M., Moradi Z., Mehrvar M. (2024). Virome analysis of potyvirus populations infecting saffron in Iran: The discovery of a novel potyvirus. Eur. J. Plant Pathol..

[19] Ágoston J., Almási A., Pinczés D., Sáray R., Salánki K., Palkovics L. (2024). First report of saffron latent virus in *Crocus sativus* from Hungary. Plant Dis..

[20] Martinez-Fajardo C., Navarro-Simarro P., Morote L., Rubio-Moraga Á., Mondéjar-López M., Niza E., Argandoña J., Ahrazem O., Gómez-Gómez L., López-Jiménez A.J. (2024). Exploring the viral landscape of saffron through metatranscriptomic analysis. Virus Res..

[21] Atefeh Hosseini S., Julian C., Galzi S., Filloux D., Roumagnac P. (2025). First report of saffron-associated mastrevirus 1 from saffron in Iran. Plant Dis..

[22] Valouzi H., Dizadji A., Golnaraghi A., Salami S., Selmi I., Fontdevila Pareta N., Önder S., Massart S. (2025). First detection of saffron dwarf virus, wheat dwarf virus, wheat dwarf virus-associated alphasatellite and a new putative potyvirus species in saffron in Iran. New Dis. Rep..

[23] Tavoosi M., Moradi Z., Mehrvar M., Zakiaghl M. (2025). First identification and complete genomic characterization of saffron dwarf virus from Iran, a novel mastrevirus infecting *Crocus sativus*. Eur. J. Plant Pathol..

[24] Parizad S., Dizadji A., Habibi M.K., Winter S., Kalantari S., Movi S., Tendero C.L., Alonso G.L., Moratalla-Lopez N. (2019). The effects of geographical origin and virus infection on the saffron (*Crocus sativus* L.) quality. Food Chem..

[25] Moratalla-López N., Parizad S., Habibi M.K., Winter S., Kalantari S., Bera S., Lorenzo C., García-Rodríguez M.V., Dizadji A., Alonso G.L. (2021). Impact of two different dehydration methods on saffron quality, concerning the prevalence of Saffron latent virus (SaLV) in Iran. Food Chem..

[26] Shamshiri M., Sánchez C., Rico S., Mokhtassi-Bidgoli A., Ayyari M., Rezadoost H., Shams-Bakhsh M. (2025). Molecular, Metabolic, and Physiological Responses to Progressive Biotic Stress Caused by Cucumber Mosaic Virus and Turnip Mosaic Virus in Saffron. Horticulturae.

[27] MacDiarmid R., Rodoni B., Melcher U., Ochoa-Corona F., Roossinck M. (2013). Biosecurity implications of new technology and discovery in plant virus research. PLoS Pathog..

[28] Muluneh M.G. (2021). Impact of climate change on biodiversity and food security: A global perspective—A review article. Agric. Food Secur..

[29] Massart S., Olmos A., Jijakli H., Candresse T. (2014). Current impact and future directions of high throughput sequencing in plant virus diagnostics. Virus Res..

[30] Al Rwahnih M., Daubert S., Golino D., Islas C., Rowhani A. (2015). Comparison of next-generation sequencing versus biological indexing for the optimal detection of viral pathogens in grapevine. Phytopathology.

[31] Hou W., Li S., Massart S. (2020). Is there a “biological desert” with the discovery of new plant viruses? A retrospective analysis for new fruit tree viruses. Front. Microbiol..

[32] Temple C., Blouin A.G., De Jonghe K., Foucart Y., Botermans M., Westenberg M., Schoen R., Gentit P., Visage M., Verdin E. (2022). Biological and genetic characterization of Physostegia chlorotic mottle virus in Europe based on host range, location, and time. Plant Dis..

[33] Rivarez M.P.S., Pecman A., Bačnik K., Maksimović O., Vučurović A., Seljak G., Mehle N., Gutiérrez-Aguirre I., Ravnikar M., Kutnjak D. (2023). In-depth study of tomato and weed viromes reveals undiscovered plant virus diversity in an agroecosystem. Microbiome.

[34] Roossinck M.J., Martin D.P., Roumagnac P. (2015). Plant virus metagenomics: Advances in virus discovery. Phytopathology.

[35] Maclot F., Debue V., Malmstrom C.M., Filloux D., Roumagnac P., Eck M., Tamisier L., Blouin A.G., Candresse T., Massart S. (2023). Long-term anthropogenic management and associated loss of plant diversity deeply impact virome richness and composition of Poaceae communities. Microbiol. Spectr..

[36] Olmos A., Boonham N., Candresse T., Gentit P., Giovani B., Kutnjak D., Liefting L., Maree H., Minafra A., Moreira A. (2018). High-throughput sequencing technologies for plant pest diagnosis: Challenges and opportunities. EPPO Bull..

[37] Licciardello G., Ferraro R., Scuderi G., Russo M., Catara A.F. (2021). A simulation of the use of high throughput sequencing as pre-screening assay to enhance the surveillance of citrus viruses and viroids in the EPPO region. Agriculture.

[38] Claverie S., Varsani A., Hoareau M., Filloux D., Roumagnac P., Martin D.P., Lefeuvre P., Lett J.-M. (2020). Sorghum mastrevirus-associated alphasatellites: New geminialphasatellites associated with an African streak mastrevirus infecting wild Poaceae plants on Reunion Island. Arch. Virol..

[39] Maclot F., Candresse T., Filloux D., Malmstrom C.M., Roumagnac P., Van der Vlugt R., Massart S. (2020). Illuminating an ecological blackbox: Using high throughput sequencing to characterize the plant virome across scales. Front. Microbiol..

[40] Kutnjak D., Tamisier L., Adams I., Boonham N., Candresse T., Chiumenti M., De Jonghe K., Kreuze J.F., Lefebvre M., Silva G. (2021). A primer on the analysis of high-throughput sequencing data for detection of plant viruses. Microorganisms.

[41] Moubset O., François S., Maclot F., Palanga E., Julian C., Claude L., Fernandez E., Rott P., Daugrois J.-H., Antoine-Lorquin A. (2022). Virion-associated nucleic acid-based metagenomics: A decade of advances in molecular characterization of plant viruses. Phytopathology.

[42] Claverie S., Ouattara A., Hoareau M., Filloux D., Varsani A., Roumagnac P., Martin D.P., Lett J.-M., Lefeuvre P. (2019). Exploring the diversity of Poaceae-infecting mastreviruses on Reunion Island using a viral metagenomics-based approach. Sci. Rep..

[43] Poutaraud A., Desbiez C., Lemaire O., Lecoq H., Herrbach E. (2004). Characterisation of a new potyvirus species infecting meadow saffron Colchicum autumnale. Arch. Virol..

[44] Massart S., Lebas B., Chabirand A., Chappé A.M., Dreo T., Faggioli F., Harrison C., Macarthur R., Mehle N., Mezzalama M. (2022). Guidelines for improving statistical analyses of validation datasets for plant pest diagnostic tests. EPPO Bull..

[45] Maclot F.J., Debue V., Blouin A.G., Pareta N.F., Tamisier L., Filloux D., Massart S. (2021). Identification, molecular and biological characterization of two novel secovirids in wild grass species in Belgium. Virus Res..

[46] Rong W., Rollin J., Hanafi M., Roux N., Massart S. (2023). Validation of high-throughput sequencing as virus indexing test for Musa germplasm: Performance criteria evaluation and contamination monitoring using an alien control. Phyto. Front..

[47] Palanga E., Filloux D., Martin D.P., Fernandez E., Gargani D., Ferdinand R., Zabré J., Bouda Z., Neya J.B., Sawadogo M. (2016). Metagenomic-based screening and molecular characterization of cowpea-infecting viruses in Burkina Faso. PLoS ONE.

[48] François S., Filloux D., Fernandez E., Ogliastro M., Roumagnac P. (2018). Viral metagenomics approaches for high-resolution screening of multiplexed arthropod and plant viral communities. Viral Metagenomics: Methods and Protocols.

[49] Lebas B., Adams I., Al Rwahnih M., Baeyen S., Bilodeau G.J., Blouin A.G., Boonham N., Candresse T., Chandelier A., De Jonghe K. (2022). Facilitating the adoption of high-throughput sequencing technologies as a plant pest diagnostic test in laboratories: A step-by-step description. EPPO Bull..

[50] Rollin J. (2020). Demultiplexing: Diagnostic Existing Demultiplexing Issue in Metagenomic Virus. GitHub. https://github.com/johrollin/demultiplexing.

[51] Altschul S.F., Madden T.L., Schäffer A.A., Zhang J., Zhang Z., Miller W., Lipman D.J. (1997). Gapped BLAST and PSI-BLAST: A new generation of protein database search programs. Nucleic Acids Res..

[52] Wood D.E., Salzberg S.L. (2014). Kraken: Ultrafast metagenomic sequence classification using exact alignments. Genome Biol..

[53] Afgan E., Baker D., Batut B., Van Den Beek M., Bouvier D., Čech M., Chilton J., Clements D., Coraor N., Grüning B.A. (2018). The Galaxy platform for accessible, reproducible and collaborative biomedical analyses: 2018 update. Nucleic Acids Res..

[54] Tamura K., Stecher G., Kumar S. (2021). MEGA11: Molecular evolutionary genetics analysis version 11. Mol. Biol. Evol..

[55] Letunic I., Bork P. (2021). Interactive Tree Of Life (iTOL) v5: An online tool for phylogenetic tree display and annotation. Nucleic Acids Res..

[56] Nyirakanani C., Tamisier L., Bizimana J.P., Rollin J., Nduwumuremyi A., Bigirimana V.d.P., Selmi I., Lasois L., Vanderschuren H., Massart S. (2023). Going beyond consensus genome sequences: An innovative SNP-based methodology reconstructs different Ugandan cassava brown streak virus haplotypes at a nationwide scale in Rwanda. Virus Evol..

[57] Nelson C.W., Moncla L.H., Hughes A.L. (2015). SNPGenie: Estimating evolutionary parameters to detect natural selection using pooled next-generation sequencing data. Bioinformatics.

[58] Farzadfar S., Tomitaka Y., Ikematsu M., Golnaraghi A.R., Pourrahim R., Ohshima K. (2009). Molecular characterisation of Turnip mosaic virus isolates from Brassicaceae weeds. Eur. J. Plant Pathol..

[59] Parizad S., Dizadji A., Habibi M.K., Winter S., Kalantari S., Garcıa-Arenal F., Ayllón M. (2017). Prevalence of saffron latent virus (SaLV), a new Potyvirus species, in saffron fields of Iran. J. Plant Pathol..

[60] Keremane M., Singh K., Ramadugu C., Krueger R.R., Skaggs T.H. (2024). Next generation sequencing, and development of a pipeline as a tool for the detection and discovery of citrus pathogens to facilitate safer germplasm exchange. Plants.

[61] Parizipour M.H.G., Schubert J., Behjatnia S.A.A., Afsharifar A., Habekuß A., Wu B. (2017). Phylogenetic analysis of Wheat dwarf virus isolates from Iran. Virus Genes.

[62] Köklü G., Ramsell J.N., Kvarnheden A. (2007). The complete genome sequence for a Turkish isolate of Wheat dwarf virus (WDV) from barley confirms the presence of two distinct WDV strains. Virus Genes.

[63] Huang A., Svanella-Dumas L., Vitry C., Marais A., Faure C., Candresse T. (2024). A new geminialphasatellite associated with wheat dwarf virus identified in winter barley in France. Arch. Virol..

[64] Briddon R.W., Martin D.P., Roumagnac P., Navas-Castillo J., Fiallo-Olivé E., Moriones E., Lett J.-M., Zerbini F.M., Varsani A. (2018). Alphasatellitidae: A new family with two subfamilies for the classification of geminivirus-and nanovirus-associated alphasatellites. Arch. Virol..

[65] Inoue-Nagata A.K., Jordan R., Kreuze J., Li F., López-Moya J.J., Mäkinen K., Ohshima K., Wylie S.J., Consortium I.R. (2022). ICTV virus taxonomy profile: Potyviridae 2022. J. Gen. Virol..

[66] Ho S., Fukagawa H., Gibbs A., Golnaraghi A., Ikematsu M., Katis N., Korkmaz S., Ohshima K., Soda H., Yasaka R. (2017). The Timescale of Emergence and Spread of Turnip Mosaic Potyvirus. Sci. Rep..

[67] Ohshima K., Mitoma S., Gibbs A.J. (2018). The genetic diversity of narcissus viruses related to turnip mosaic virus blur arbitrary boundaries used to discriminate potyvirus species. PLoS ONE.

[68] Zhang B., Li Q., Hu J., Zhang L., Dong X., Ji P., Dong J. (2023). Complete genome sequence analysis of a new potyvirus isolated from Paris polyphylla var. yunnanensis. Arch. Virol..

[69] de Vries J.J., Brown J.R., Couto N., Beer M., Le Mercier P., Sidorov I., Papa A., Fischer N., Oude Munnink B.B., Rodriquez C. (2021). Recommendations for the introduction of metagenomic next-generation sequencing in clinical virology, part II: Bioinformatic analysis and reporting. J. Clin. Virol..

[70] López-Labrador F.X., Brown J.R., Fischer N., Harvala H., Van Boheemen S., Cinek O., Sayiner A., Madsen T.V., Auvinen E., Kufner V. (2021). Recommendations for the introduction of metagenomic high-throughput sequencing in clinical virology, part I: Wet lab procedure. J. Clin. Virol..

[71] Nizamani M.M., Zhang Q., Muhae-Ud-Din G., Wang Y. (2023). High-throughput sequencing in plant disease management: A comprehensive review of benefits, challenges, and future perspectives. Phytopathol. Res..

[72] François S., Bernardo P., Filloux D., Roumagnac P., Yaverkovski N., Froissart R., Ogliastro M. (2014). A novel itera-like densovirus isolated by viral metagenomics from the sea barley Hordeum marinum. Genome Announc..

[73] Filloux D., Fernandez E., Comstock J.C., Mollov D., Roumagnac P., Rott P. (2018). Viral metagenomic-based screening of sugarcane from Florida reveals occurrence of six sugarcane-infecting viruses and high prevalence of Sugarcane yellow leaf virus. Plant Dis..

[74] Schönegger D., Moubset O., Margaria P., Menzel W., Winter S., Roumagnac P., Marais A., Candresse T. (2023). Benchmarking of virome metagenomic analysis approaches using a large, 60+ members, viral synthetic community. J. Virol..

[75] Bernardo P., Charles-Dominique T., Barakat M., Ortet P., Fernandez E., Filloux D., Hartnady P., Rebelo T.A., Cousins S.R., Mesleard F. (2018). Geometagenomics illuminates the impact of agriculture on the distribution and prevalence of plant viruses at the ecosystem scale. ISME J..

[76] Ma Y., Marais A., Lefebvre M., Theil S., Svanella-Dumas L., Faure C., Candresse T. (2019). Phytovirome analysis of wild plant populations: Comparison of double-stranded RNA and virion-associated nucleic acid metagenomic approaches. J. Virol..

[77] Masangwa J.I.G., Pareta N.F., Moses P., Hřibová E., Doležel J., Fandika I., Massart S. (2024). Surveillance and molecular characterization of banana viruses and their association with Musa germplasm in Malawi. bioRxiv.

[78] Ren Y., Xu Y., Lee W.M., Di Bisceglie A.M., Fan X. (2020). In-depth serum virome analysis in patients with acute liver failure with indeterminate etiology. Arch. Virol..

[79] Gorbalenya A.E., Lauber C., Siddell S.G. (2019). Taxonomy of Viruses. Reference Module in Biomedical Sciences.

[80] Futschik A., Schlötterer C. (2010). The next generation of molecular markers from massively parallel sequencing of pooled DNA samples. Genetics.

[81] Technow F., Gerke J. (2017). Parent-progeny imputation from pooled samples for cost-efficient genotyping in plant breeding. PLoS ONE.

[82] Ko B., Van Raamsdonk J.M. (2023). RNA sequencing of pooled samples effectively identifies differentially expressed genes. Biology.

[83] Matthews A.E., Boves T.J., Percy K.L., Schelsky W.M., Wijeratne A.J. (2023). Population Genomics of Pooled Samples: Unveiling Symbiont Infrapopulation Diversity and Host–Symbiont Coevolution. Life.

[84] Tabasi M., Mehrabian A., Sayadi S. (2021). Distribution patterns and conservation status of Crocus species in Iran, one of the diversity centers of Crocus in the Middle East. Folia Oecologica.

[85] Movi S., Dizadji A., Parizad S., Zarghani S.N. (2022). Biological characteristics and genetic variation analyses of saffron latent virus (SaLV) based on genomic P1-Pro and P3 regions. Eur. J. Plant Pathol..

[86] Maachi A., Donaire L., Hernando Y., Aranda M.A. (2022). Genetic differentiation and migration fluxes of viruses from melon crops and crop edge weeds. J. Virol..

[87] Schönegger D., Marais A., Babalola B.M., Faure C., Lefebvre M., Svanella-Dumas L., Brázdová S., Candresse T. (2023). Carrot populations in France and Spain host a complex virome rich in previously uncharacterized viruses. PLoS ONE.

[88] Tavoosi M., Moradi Z., Mehrvar M. (2022). First report of Turnip mosaic virus infecting saffron in Iran. Virus Dis..

[89] Tatineni S., Hein G.L. (2023). Plant viruses of agricultural importance: Current and future perspectives of virus disease management strategies. Phytopathology.

[90] Khatun M.F., Kwak M., Kwon M., Hossain M.M., Kil E.-J. (2025). New insights into viral threats in soybean (Glycine max) crops from Bangladesh, including a novel crinivirus. Front. Microbiol..

[91] Fontdevila Pareta N., Khalili M., Maachi A., Rivarez M.P.S., Rollin J., Salavert F., Temple C., Aranda M.A., Boonham N., Botermans M. (2023). Managing the deluge of newly discovered plant viruses and viroids: An optimized scientific and regulatory framework for their characterization and risk analysis. Front. Microbiol..

[92] Lorenzo C., Shadmani G., Valouzi H., Moratalla-López N., Bahlolzada H., Sánchez-Gómez R., Dizadji A., Alonso G.L. (2023). Saffron Stigmas Apocarotenoid Contents from Saffron Latent Virus (SaLV)-Infected Plants with Different Origins and Dehydration Temperatures. Horticulturae.

[93] Navalinskienė M., Samuitienė M. (2001). Viruses affecting some bulb and corm flower crops. Biologija.

[94] Alavi-Siney S.M., Saba J., Siahpirani A.F., Nasiri J. (2023). ISSR-assisted spatial genetic structure, population admixture, and biodiversity estimates across locally adopted saffron ecotypes from 18 different provenances of Iran. J. Appl. Res. Med. Aromat. Plants.

[95] Bazoobandi M., Rahimi H., Karimi-Shahri M.R., Koocheki A., Khajeh-Hosseini M. (2020). Saffron crop protection. Saffron.

[96] Nigam D., LaTourrette K., Souza P.F., Garcia-Ruiz H. (2019). Genome-wide variation in potyviruses. Front. Plant Sci..

[97] Yang X., Li Y., Wang A. (2021). Research advances in potyviruses: From the laboratory bench to the field. Annu. Rev. Phytopathol..

