# Supplementary material for: A Virome Scanning of Saffron (Crocus sativus L.) at the National Scale in Iran Using High-Throughput Sequencing Technologies
Source: Viruses. 2025 Aug 4;17(8):1079. doi: 10.3390/v17081079 (PMC12390696; doi:10.3390/v17081079)

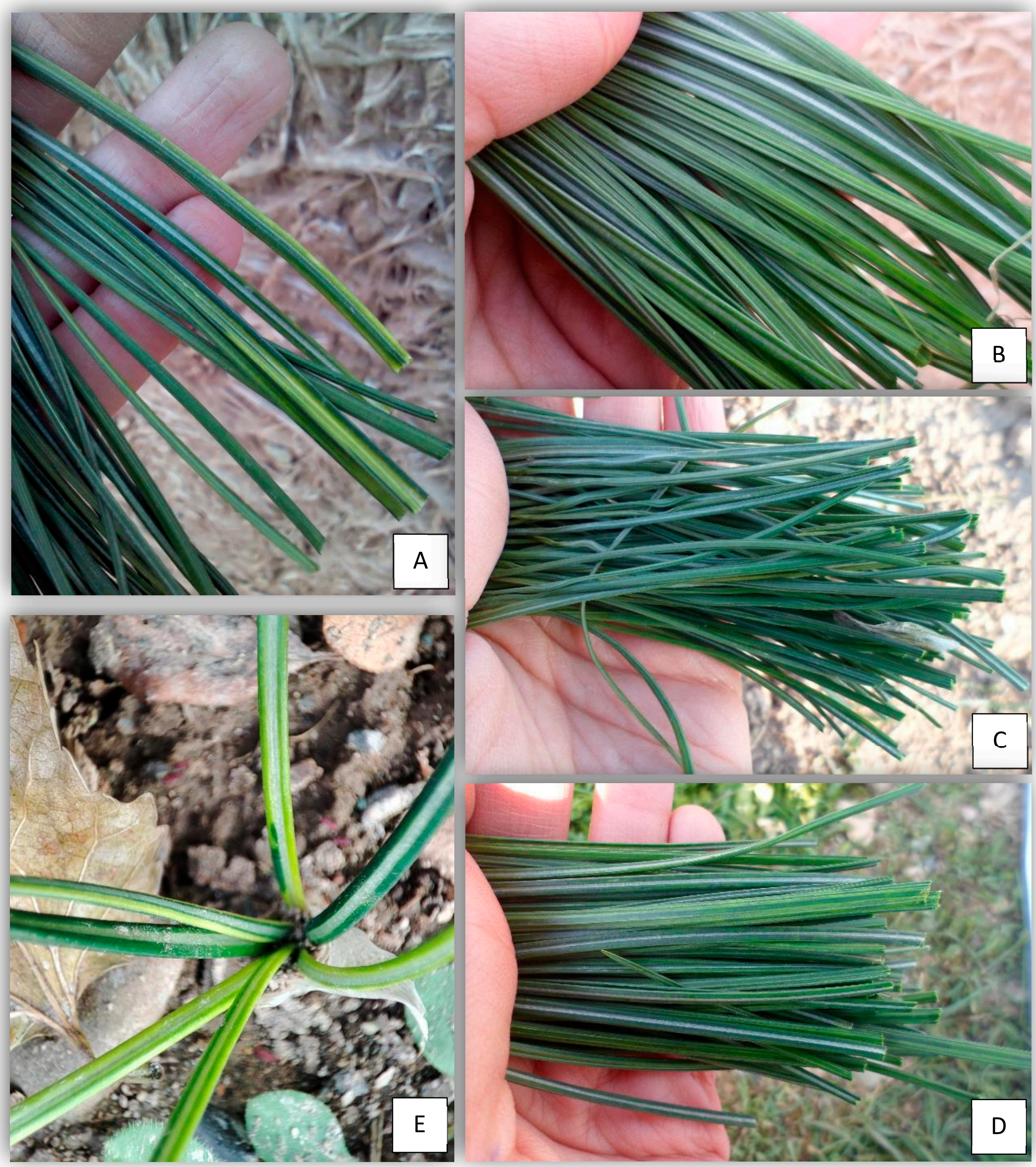

Figure S1. Symptoms of some collected plants: A. yellowing; B. mottling; C. leaf curl; D. mosaic; E. yellowing and mosaic

Samples	province	cultivation area (ha)	no. of detected viruses and satellite
H2	Qom	27	2
H6	Tehran	198	5
H4	Markazi	206	3
H5	Semnan	256.5	2
H20	Semnan	256.5	3
H1	East Azerbaijan	294	3
H3	Yazd	300	4
H17	Yazd	300	4
H11	Kerman	436	6
H16	Kerman	436	3
H19	Kerman	436	4
H8	Fars	940	5
H7	Isfahan	1137	4
H14	South Khorasan	5343.6	4
H15	South Khorasan	5343.6	3
H18	South Khorasan	5343.6	5
H10	Razavi Khorasan	28275	3
H12	Razavi Khorasan	28275	6
H13	Razavi Khorasan	28275	5

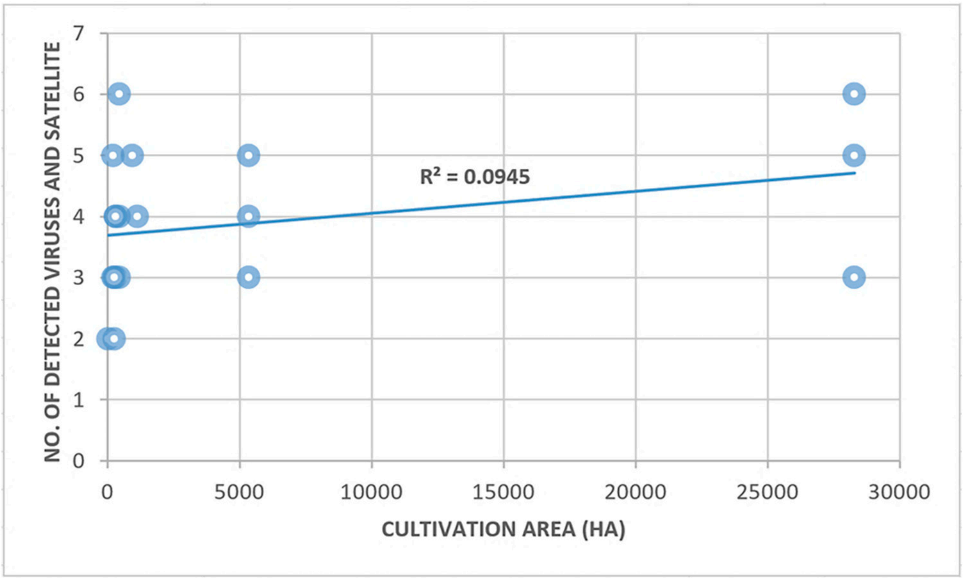

Figure S2. Table and scatter plot illustrating the relationship between the cultivation area (x-axis) and the number of detected viruses (y-axis). Each data point represents a pooled sample, and in cases where multiple pooled samples were collected from a single province, we allocated the province's total cultivation area equally among its samples. The R^2 value of the linear regression analysis indicates that only about 9.45% of the variability in the number of detected viruses can be explained by the cultivation area. Each row of the table represents a pooled sample, detailing the cultivation area (in hectares) and the number of viruses detected.

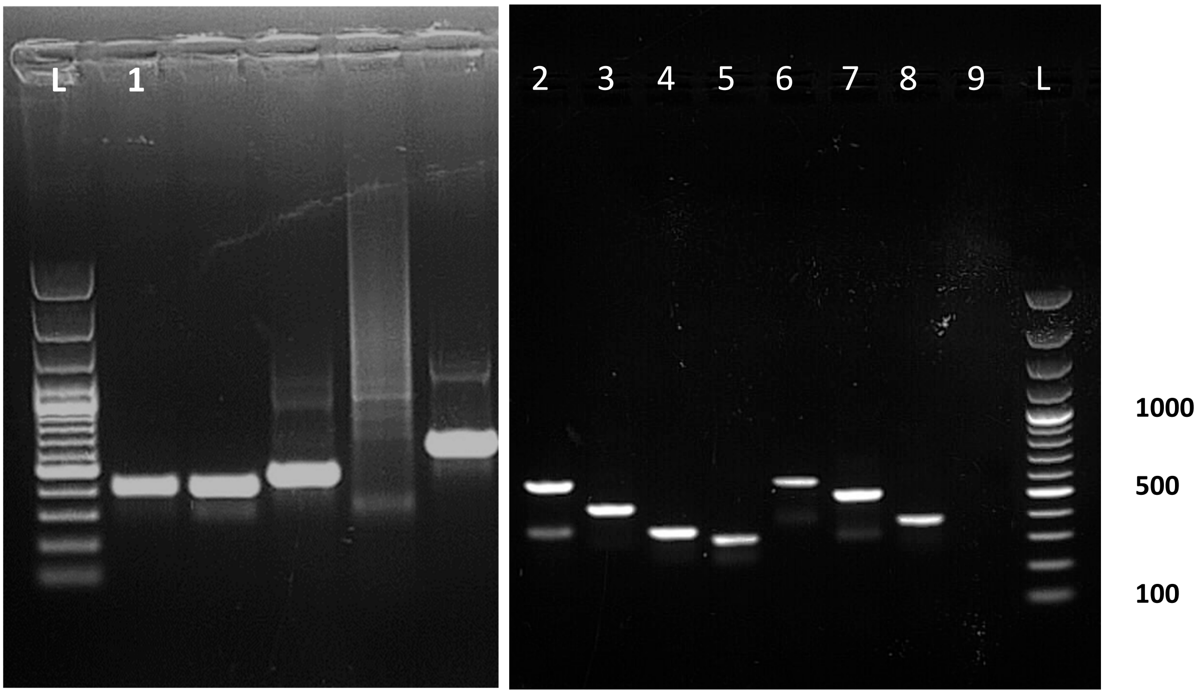

Figure S3. Electrophoresis Gel of PCR and RT-PCR products using the designed primer pairs to confirm high-throughput sequencing (HTS) results. From left to right: 1, SYMV (expected amplicon size 392 bp); 2, SaIRV (569 bp); 3, SaLV (434 bp); 4, TuMV (338 bp); 5, BWYV (307 bp); 6, WDV (576 bp); 7, SaDV (496 bp); 8, WDVaA (376 bp); 9, negative control. The ladder used was the GeneRuler 1 kb plus ladder (Thermo Fisher Scientific, SM1343). Additional wells contain samples unrelated to this study.

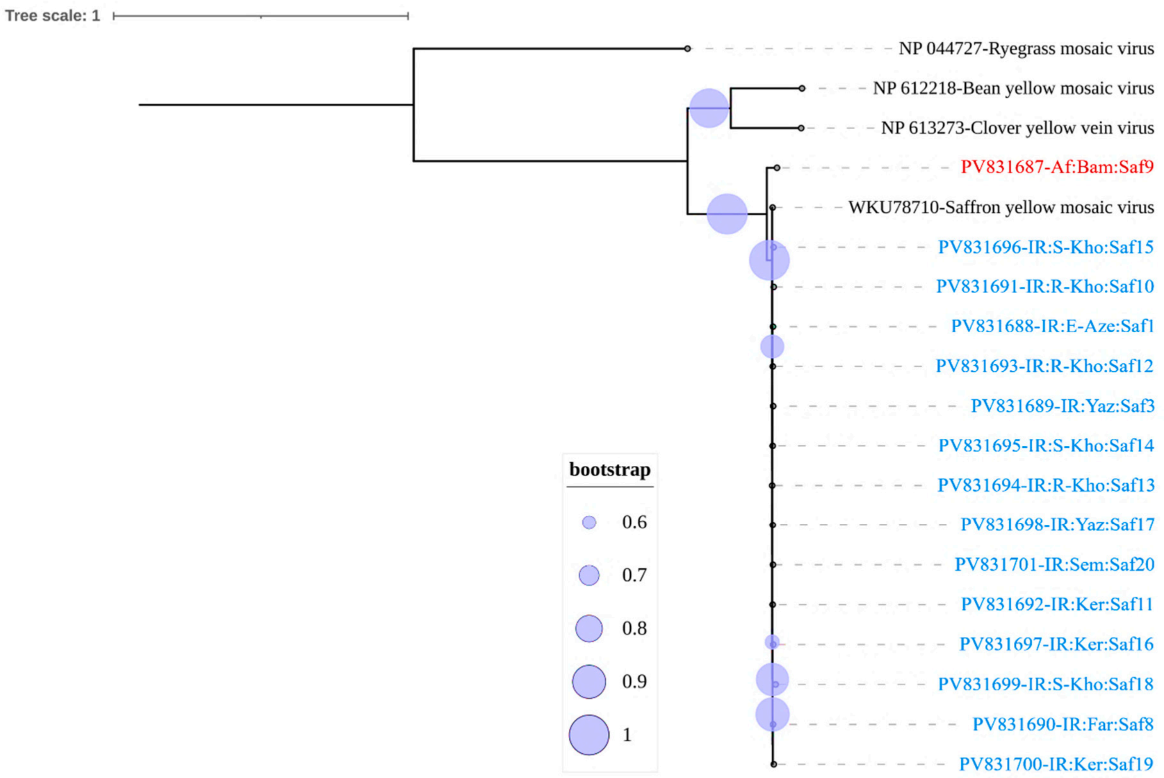

Figure S4. Maximum likelihood phylogenetic tree based on a multiple-amino-acid alignment of SYMV polyprotein sequences (using the Jones–Taylor–Thornton (JTT) model with 1000 bootstraps). The tree includes SYMV sequences discovered in this study as well as SYMV (WKU78710), BYMV (NP 612218), and ClYVV (NP 613273). Circles at the nodes indicate bootstrap support, as explained in the adjacent legend. The analysis involved 19 amino acid sequences, with a total of 3163 positions in the final dataset. The Iranian sequences are in blue, and the Afghan in red. Evolutionary analyses were conducted using MEGA11.

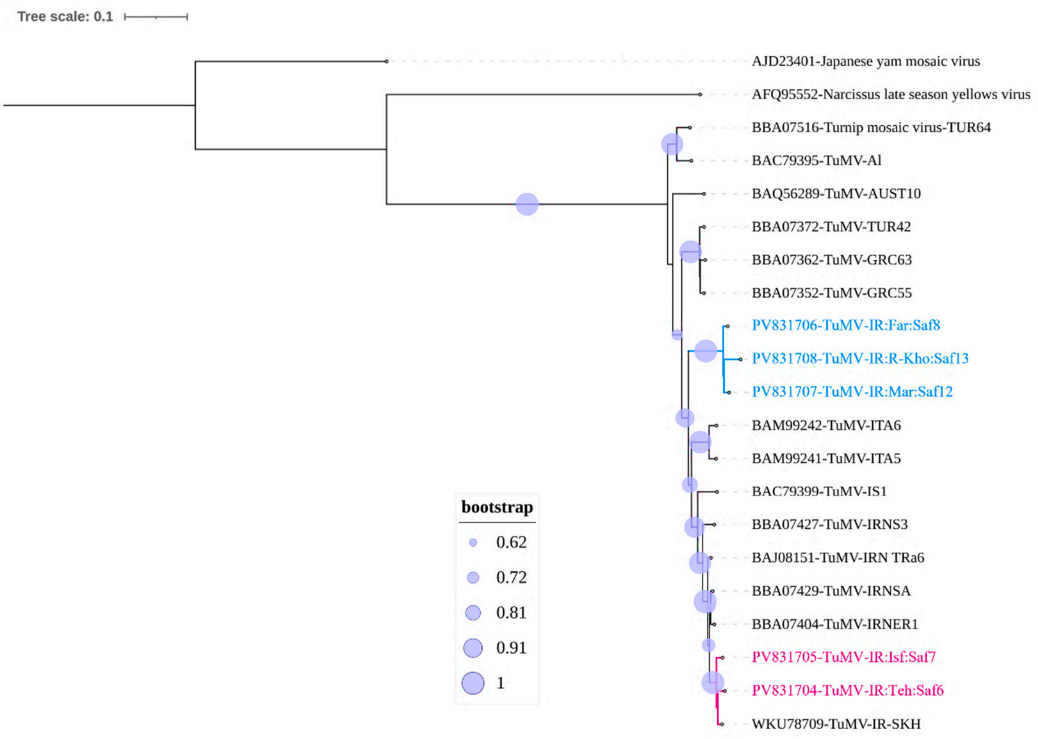

Figure S5. Maximum likelihood phylogenetic tree based on a multiple amino-acid-alignment of TuMV polyprotein sequences (using JTT model with 1000 bootstraps). The tree includes TuMV sequences discovered in this study (H06, H07, H08, H12, and H13) as well as other TuMV isolates. Narcissus late season yellows virus (AFQ95552) was used as an outgroup. Circles at the nodes indicate bootstrap support, as explained in the adjacent legend. The analysis involved 21 amino acid sequences, with a total of 3170 positions in the final dataset. Evolutionary analyses were conducted using MEGA11.

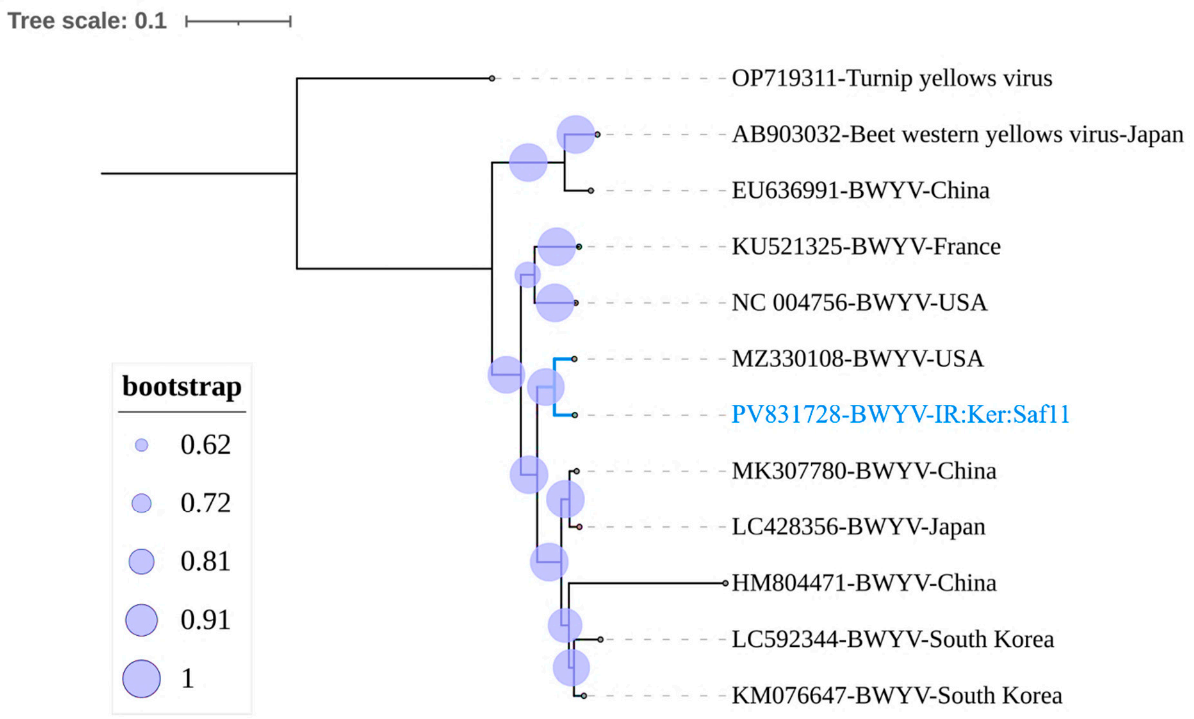

Figure S6. Maximum likelihood phylogenetic tree based on a multiple-nucleotide alignment of BWYV full-length genome sequences (using the Tamura–Nei model with 1000 bootstraps). The tree includes the beet western yellows virus (BWYV) discovered in this study and other isolates of BWYV. Turnip yellows virus was used as an outgroup. Circles at the nodes indicate bootstrap support, as explained in the adjacent legend. This analysis involved 12 nucleic acid sequences. There were a total of 5787 positions in the final dataset. Evolutionary analyses were conducted using MEGA11.

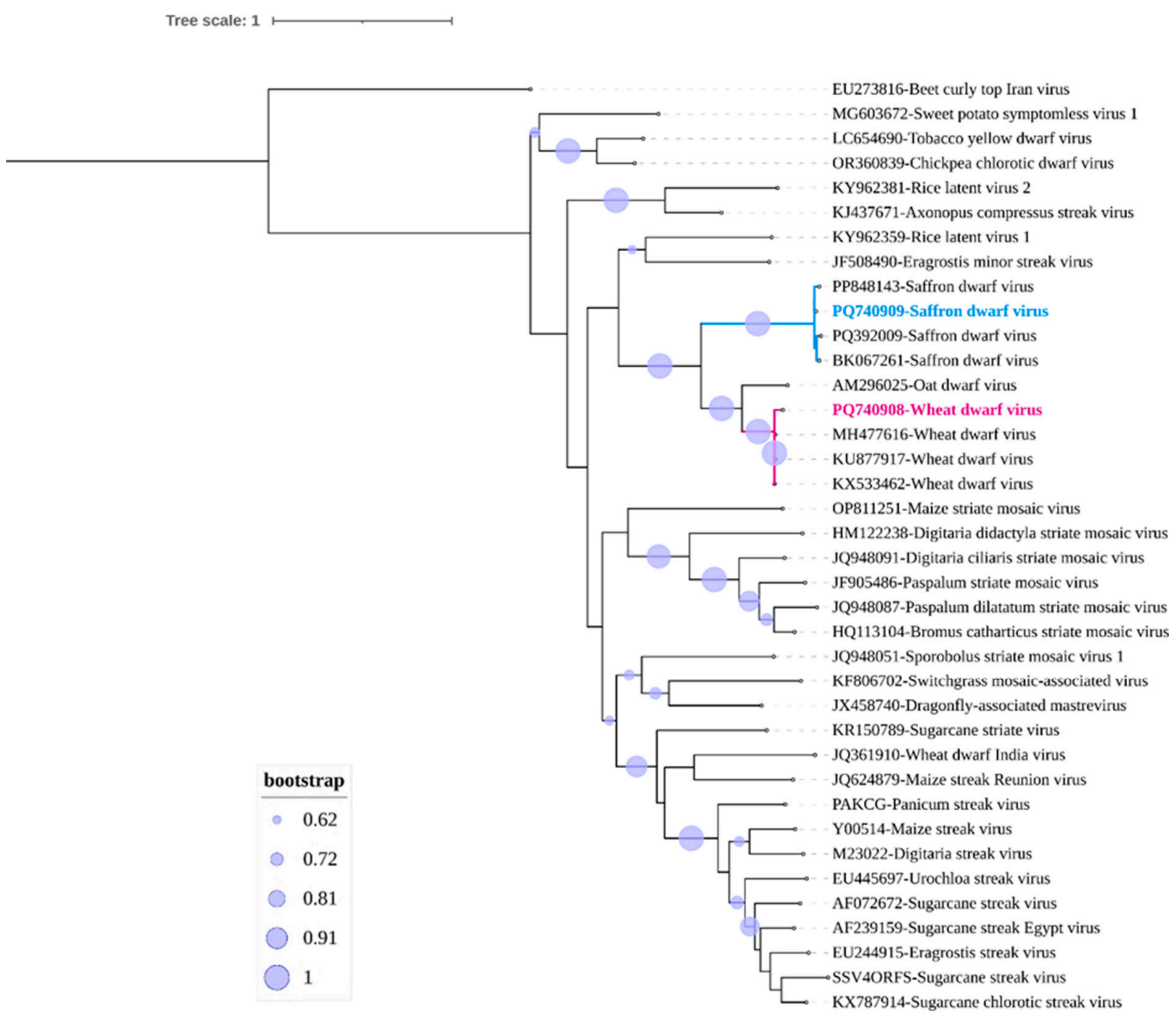

Figure S7. Maximum likelihood phylogenetic tree based on a multiple-amino-acid alignment of *Mastrevirus* replicase protein sequences (using the Le_Gascuel_2008 model with 1000 bootstraps). The tree includes the saffron dwarf virus (SaDV) and wheat dwarf virus (WDV) discovered in this study and other members of the *Mastrevirus* genus. Beet curly top Iran virus from *Becurtovirus* was used as an outgroup. Viral sequences identified in this study are highlighted in color. Circles at the nodes indicate bootstrap support, as explained in the adjacent legend. This analysis involved 38 amino acid sequences. There were a total of 415 positions in the final dataset. Evolutionary analyses were conducted using MEGA11.

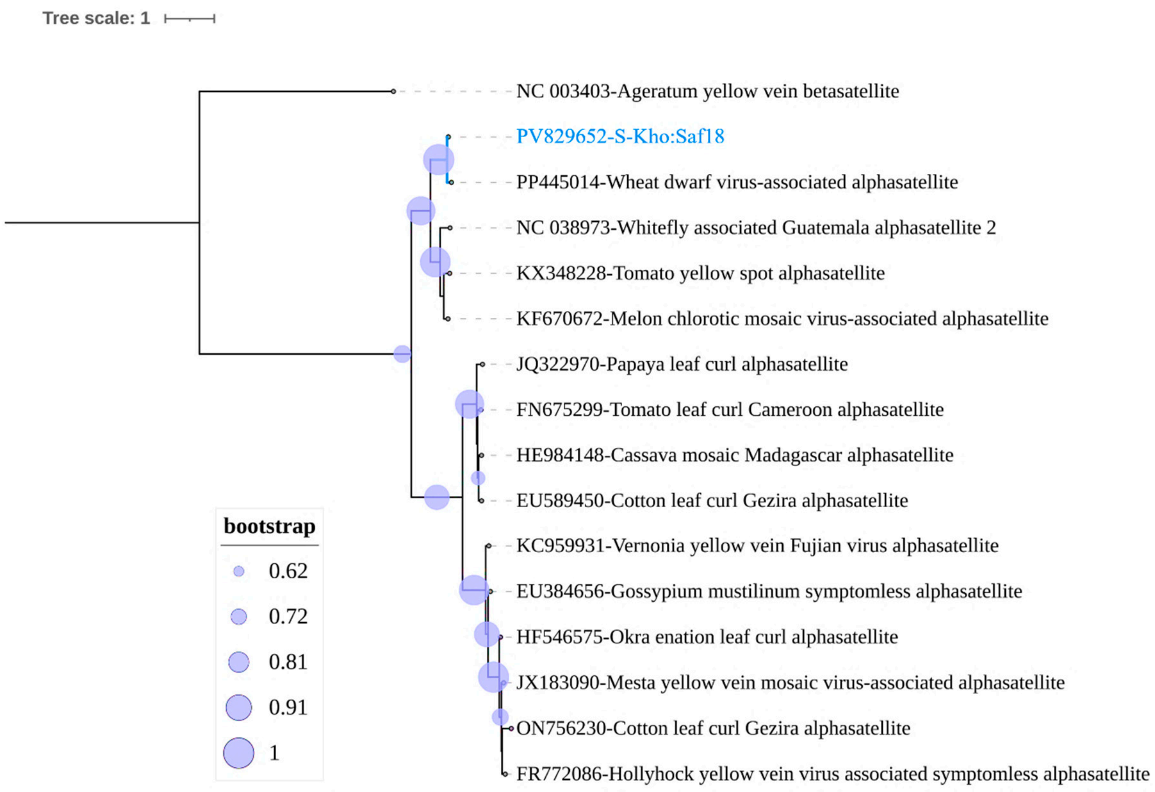

Figure S8. Maximum likelihood phylogenetic tree based on a multiple-amino-acid alignment of Alphasatellite replicase protein sequences (using JTT model with 1000 bootstraps). The tree includes the Alphasatellite discovered in this study and other members of the Alphasallitidae family. Ageratum yellow vein betasatellite was used as an outgroup. The Alphasatellite sequence identified in this study is highlighted in color. Circles at the nodes indicate bootstrap support, as explained in the adjacent legend. This analysis involved 16 amino acid sequences. There were a total of 329 positions in the final dataset. Evolutionary analyses were conducted using MEGA11.

Figure S9 (next page). Visualization of the virome network for the virus species detected in saffron samples from various provinces of Iran. a. Province view: Samples from different provinces remain in fixed positions, showing how viruses are distributed across the network. b. Virus view: Viruses are positioned at the center, with samples from different provinces organized around them. The colors represent different regions of Iran: red (west), blue (center), green (south), and orange (east).

a. Province-centered view

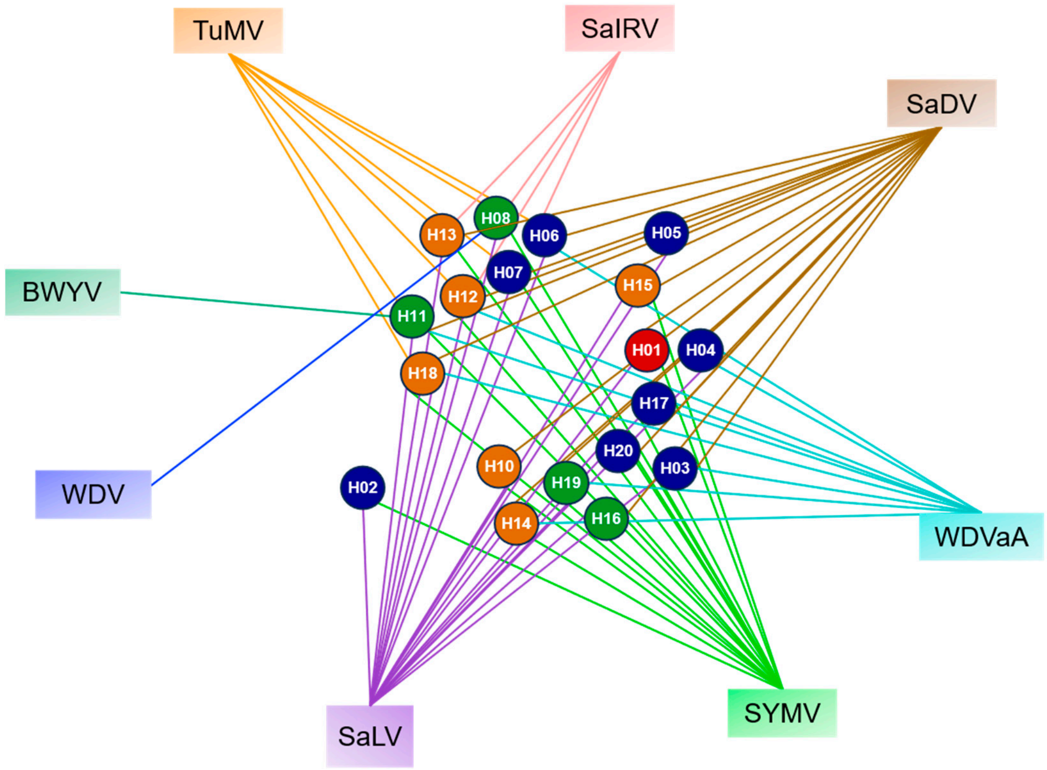

b. Virus-centered view

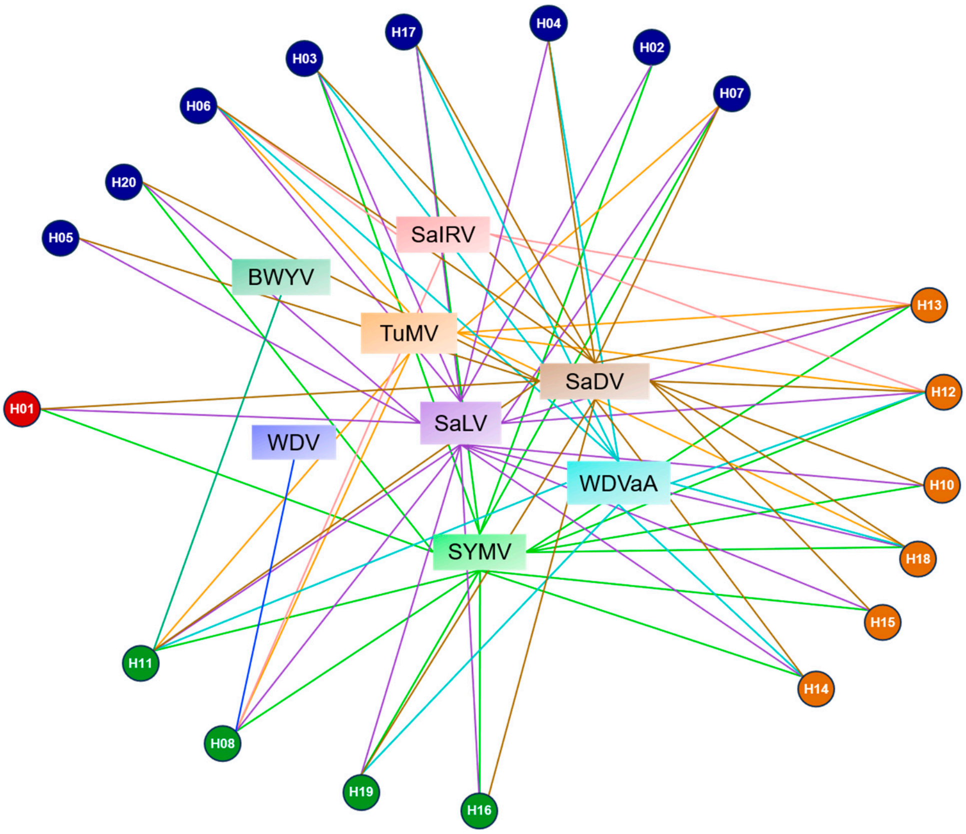

Supplement: Supplementary file 1 [file viruses-17-01079-s001.zip › Supplimantaries/Supplementary figures.pdf]
